# Veteran deployed burn pits exposure: headache phenotypes, trajectories, and outcomes: a mixed-methods evaluation

**DOI:** 10.3389/fneur.2026.1899580

**Published:** 2026-10-22

**Authors:** Cassandra Laferriere, Daniel G. Rogers, Nicholas A. Rattray, Roberta E. Goldman, Sean A. Baird, Laura Burrone, Katrina Spontak, Jason J. Sico, Teresa M. Damush

**Affiliations:** 1 Headache Centers of Excellence, Veterans Affairs, VA Connecticut Healthcare System, West Haven, CT, United States; 2 Department of Neurology, Yale School of Medicine, New Haven, CT, United States; 3 VA HSR Center for Health Information and Communication (CHIC), Richard L. Roudebush VA Medical Center, Indianapolis, IN, United States; 4 Division of General Internal Medicine and Geriatrics, Department of Medicine, Indiana University School of Medicine, Indianapolis, IN, United States; 5 Regenstrief Institute, Indianapolis, IN, United States; 6 Department of Family Medicine, Alpert Medical School of Brown University, Providence, RI, United States

**Keywords:** headache, migraine, mixed-methods, open burn pit exposure, phenotypes, post-traumatic headache

## Abstract

**Background:**

Exposure to open burn pit smoke and fumes during military service has been associated with a range of chronic health conditions. Little is known about the specific phenotypical manifestations of open burn pit-related headaches and their health impacts.

**Aims:**

The study aimed to: 1. classify headache phenotypes and evaluate associated health outcomes among Veterans with self-reported open burn pit exposure and headache; 2. use a mixed-methods approach to characterize broader health impacts by exposure level and headache phenotype; and 3. identify healthcare trajectories of Veterans exposed to burn pits during and after deployment, including satisfaction with headache care within the Veterans Health Administration (VHA).

**Methods:**

We analyzed a sample of 247,583 participants with linked self-reported open burn pit exposure and International Classification of Diseases (ICD)-coded health data from the Airborne Hazards and Open Burn Pit (AH&OBP) Registry, Department of Defense (DoD), and VHA Electronic Health Records (EHRs). A randomly selected subset of 1,980 participants received an online) survey assessing sociodemographic information, burn pit exposure, headache symptoms and outcomes, care seeking, social determinants of health (SDOH), and acceptability of care. Respondents were invited to participate in semi-structured interviews.

**Results:**

Among the 772 Veterans who responded (39.0%), 335 (43.4%) completed all headache phenotyping items. We conducted interviews with 49 Veterans. Quantitative findings showed significant differences across headache phenotypes in headache-related disability and insomnia. Veterans with migraine and post-traumatic headache (PTH) reported greater impairment than those with “other headache”, suggesting higher overall disease burden relative to other headache types. Additionally, 68.0% of Veterans who completed SDOH items reported social isolation. Qualitative findings further contextualized symptom burden within Veterans lived experiences. Many described onset of headache symptoms during deployment and inconsistent access to guideline-concordant headache care. Post-deployment trajectories varied widely, particularly in coordination, continuity, and access to stepped headache care (SHC).

**Conclusions:**

Veterans exposed to open burn pits exhibited heterogenous post-deployment healthcare trajectories and high disease burden, particularly among those with migraine and PTH. Improving care coordination, integrating patient navigation services, and prioritizing outreach to socially isolated Veterans may improve headache management and reduce long-term disability.

## Introduction

Airborne hazards refer to contamination from potentially toxic substances that people are exposed to through the air they breathe (1). Military service members may be exposed to many such hazards while on deployment, such as sand and dust, fuel and aircraft exhaust, and smoke and fumes from open burn pits (1). Open burn pit smoke and fumes, a specific airborne hazard, were common during United States (US) military operations in Iraq, Afghanistan, Syria and other areas of the Middle East and Southwest Asia theater of military operations (1). In 2013, the US Congress passed Public Law 112-260 (2) which directed the Veterans Affairs (VA) agency to create the Airborne Hazards and Open Burn Pit (AH&OBP) Registry to monitor the health effects from open burn pit exposure, and ultimately, treatment for Veterans (3). According to the US Department of Defense (DoD), approximately 4.7 million Veterans and service members are eligible for inclusion in the registry (4).

Among US military personnel and Veterans, exposure to open burn pit smoke and fumes during military service has been associated with a range of chronic health conditions, including increased risk of developing respiratory diseases and hypertension (5). Recent evidence suggests that open burn pit exposure during military deployment is associated with increased odds of medically diagnosed headache disorders. Participants in the highest open burn pit exposure group had 59% higher adjusted odds of medically diagnosed headache and 60% higher adjusted odds of medically diagnosed migraine than participants without open burn pit exposure (6). Moreover, an association was identified between the amount of open burn pit exposure (as cumulative number of exposure days) and being diagnosed with a new headache disease by a clinical provider, such that those in the highest bracket of exposure had a 55% higher adjusted odds of developing headache and 48% of developing migraine than those with no burn pit exposure (6). However, less is known about the specific phenotypical manifestations of Open Burn Pit Exposure-related Headaches and how open burn pit exposures may influence headache-related outcomes.

Though emerging evidence points to a relationship between burn pit exposure and headache (6), there remains a paucity of data characterizing headache phenotypes among those with burn pit exposure and headache. To our knowledge, there has been no exploration of how Veterans navigate headache care within a given military healthcare system or as they transition to post-deployment and into the Veterans Health Administration (VHA) and/or community.

In this mixed-methods evaluation, we applied an adapted version of the Stepped Care Model for Chronic Pain Management (SCM-PM) to evaluate Veterans navigation through the VHA (7). The VHA introduced SCM-PM in 2009 as an essential part of the VHA National Pain Management Strategy (NPMS) (8). The SCM-PM emphasizes equitable access to health care, triage of treatment based on patient needs, and the use of resources for preventive care. Figure 1 presents the adapted SCM-PM framework we utilized for identifying headache care received across the trajectory of military deployment accompanied by open burn pit exposures. Similar to chronic pain management, foundational headache care is based at the patient level of self-care and the family level through Whole Health principles of patient-driven care which focuses on the biopsychosocial model rather than a single symptom or disease (9). The progression of stepped care involves: 1. primary care management of common headache symptoms; 2. timely referral to specialty care consultation (i.e., Neurology) with coordination at a regional level and with clinical resource networks; and 3. advanced specialty headache care (8). Concurrently, the model includes the assessment and addressing of social determinants of health (SDOH) across the steps as promoted in the Whole Health system of care in VHA, which has not been routinely implemented (10, 11).

**Figure 1:**
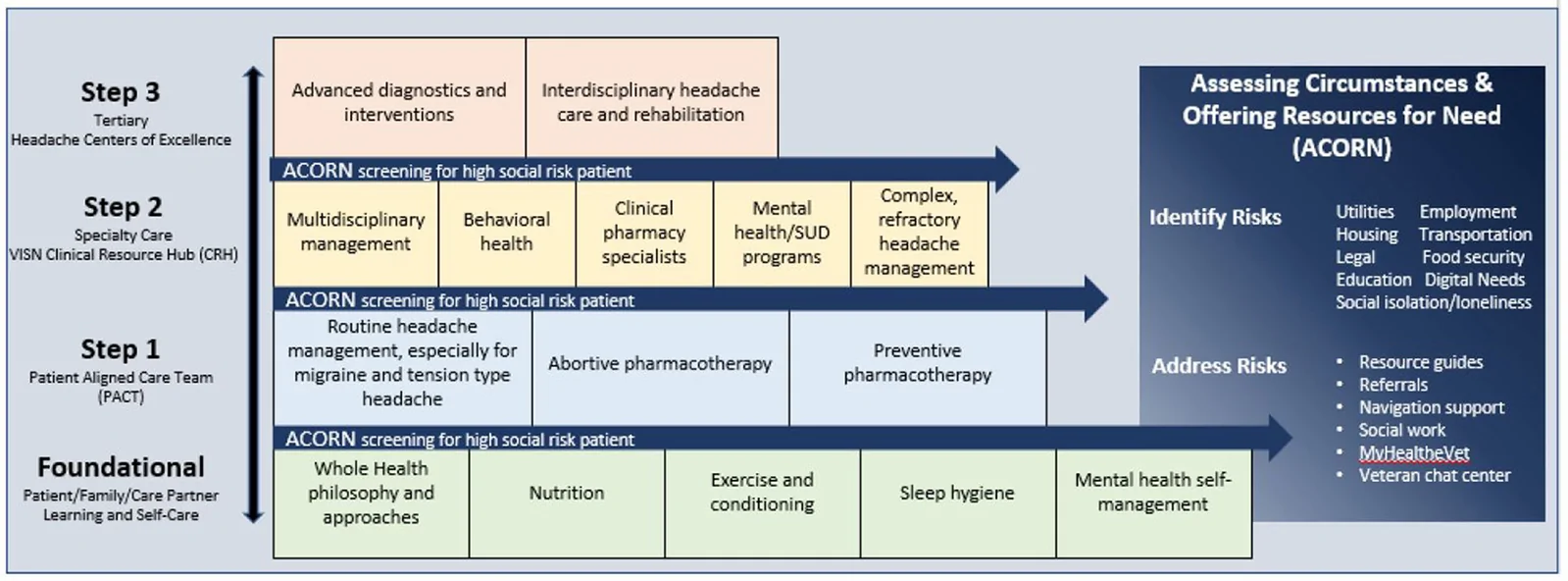
Stepped care: headache disease management.

Among Veterans with self-reported open burn pit exposure and headache, we sought to characterize their headache disease phenotype (e.g., migraine, post-traumatic headache [PTH]) and to understand the broader health impacts of open burn pit exposure. The specific aims were three-fold: 1. To classify headache phenotypes and to evaluate the associated health outcomes of open burn pit exposure among Veterans with self-reported open burn pit exposure and headache; 2. To conduct a mixed-methods analysis of the broader health impacts of open burn pit exposure by exposures and by headache phenotypes across deployment; and 3. To identify the healthcare trajectory of Veterans exposed to open burn pits during deployment and experiencing headaches from active duty and continued post-deployment as care is transitioned into the Department of Veteran Affairs Medical Centers across Stepped Headache Care (SHC), and evaluate Veterans' satisfaction with their headache care.

## Materials and methods

This study utilized a data set combining the AH&OBP Registry data in conjunction with data from the US DoD and the VHA (6) to identify Veterans with headache and those with and without open burn pit exposure. From this data set, participants were selected using stratified random sampling based on exposure status. To evaluate the impact of proximity and related duties on headache condition in greater detail, we compiled a comprehensive survey followed by a virtual interview with a subset of the survey participants. Thus, we conducted an explanatory sequential study where we followed the quantified survey with a qualitative interview to gain insight and further expand upon the survey data.

The study was approved by the VA Connecticut Healthcare System (VACHS) Institutional Review Board (IRB) with a waiver for written informed consent as the risk was determined to be minimal for participants. Veterans who voluntarily completed a Research Electronic Data Capture (REDCap) survey after reading the study information sheet provided implied consent. We recorded informed consent verbally from the interview participants prior to beginning the virtual interview.

## Survey eligibility

We utilized a previous analytic sample of 247,583 participants with linked self-reported open burn pit exposure during a US military deployment. Data from the AH&OBP Registry were linked with the VA Corporate Data Warehouse (CDW) for VHA Electronic Health Records (EHRs) and the Department of Defense VA Informatics and Computing Infrastructure (DaVINCI) for DoD EHRs, with ICD codes used to identify headache diagnoses (6).

Using a stratified random sampling approach, we randomly selected individuals falling into registry-defined categories of open burn pit exposure and headache status who had been active in the VHA (i.e., completed a VHA visit within the last 18 months. Registry-defined open burn pit exposure categories included veterans who reported (1) being near an open burn pit and having open burn pit duties, (2) being near an open burn pit but not having open burn pit duties, or (3) not being near an open burn pit and having no open burn pit duties. Headache statues consistent of persistent, resolved, delayed, and no headache. Eligible Veterans were identified and sent an email describing the study and given a 2-week period to opt out of participation. Remaining participants were then emailed the survey evaluating self-reported open burn pit exposure and health concerns, with an emphasis on headaches and migraine. The survey consisted of additional sociodemographic information, questions on open burn pit exposure, headache and health symptoms, headache outcomes and treatment satisfaction, care seeking, SDOH, and patients' assessment of acceptability of medical care.

## Interview eligibility

Those Veterans eligible for interview participation had to have asserted in the AH&OBP Registry Self-Assessment having been exposed to open burn pit and/or other toxins, with or without having had directly related open burn pit duties (6). Respondents were excluded if they did not report open burn pit exposure (i.e., not near open burn pit or no open burn pit duties) as well as Veterans who reported no headache symptoms and/or condition at any time in their survey response. The survey concluded by asking participants if they were willing to participate in interview.

Each semi-structured interview was conducted via Microsoft Teams with a study team member. They were conducted with Veterans on a rolling basis upon receiving survey responses, from January 2025 to May 2025. After discussing the study aims, the interviewer obtained verbal informed consent for participation and audio recording of the interview before beginning the interview. Interview topics included open burn pit and other toxic exposure, headache history and care, headache impact on life, and SDOH.

## Measurement

### Categorizing headache phenotypes

To understand whether Open Burn Pit Exposure-related Headaches could be further categorized into phenotypes, we applied the International Classification of Headache Disorders, 3rd edition (ICHD-3) (12) criteria alongside coding guidance from the American Migraine Prevalence and Prevention (AMPP) study (13). The AMPP survey has demonstrated high sensitivity of 100% and specificity of 82% in identifying migraine in population studies (14). Open burn pit exposure was determined based on one variable from the survey, which asked participants if they were exposed to open burn pits. It was a 4-level categorical variable (i.e., yes, no [no exposure group], unsure, and choose not to disclose). Participants who answered “yes” were placed in the open burn pit exposure group, those who answered “no” were placed into the no open burn pit exposure group, and those who chose “unsure” or “choose not to disclose” were excluded. Post-traumatic headache (PTH) phenotype was assigned using questionnaire items based on ICHD-3 diagnostic criteria and cluster headache derived from items on the ICHD-3 diagnostic criteria and previously validated screen instruments (12). Migraine and “other headache” was derived from the AMPP survey (13). The cluster headache questionnaire items were developed using the three-item Erwin Test for Cluster Headache, which has demonstrated good diagnostic performance, with a sensitivity of 84% (95% CI: 73–92%) and specificity of 89% (95% CI: 84–94%) (15). Post-traumatic headache was classified using questionnaire items operationalizing the ICHD-3 diagnostic criteria, including headache onset or worsening following head injury and the temporal relationship between the injury and headache onset (12). The survey inquired about multiple types and severities of headaches; however, individuals were only counted toward one headache type with the following priority: PTH, cluster, migraine, and “other headache.” For example, an individual who screened positive for PTH and met migraine criteria via the AMPP survey was classified as PTH. Individuals with PTH were further classified into primary headache phenotypes (cluster, migraine, and other) using the same strategy. Respondents were dropped from further phenotyping if they responded to the PTH screener questions as their headache being caused by anything other than a head injury.

### Headache and related outcomes

We included validated outcome measures to assess headache symptoms and related health outcomes including headache-related disability (16) (Headache Impact Test; HIT-6), insomnia (17) (Insomnia Severity Index; ISI), depression (18) (Patient Health Questionnaire; PHQ-2), and anxiety (19) (Generalized Anxiety Disorder 2-item; GAD-2). A subset of measures was included as part of a broader effort to validate measures commonly used in pain research and adopted within the VHA (20), including measures on pain severity and impact (Pain, Enjoyment of Life, and General Activity – 3 item; PEG-3) (21), pain self-efficacy (Pain Self Efficacy Questionnaire – 2 item; PSEQ) (22). A SDOH screen (Assessing Circumstances and Offering Resources for Needs [ACORN]) was also included to offer insight into the differing SDOH needs between the exposure groups (23). Finally, we included questions of Veteran satisfaction with their headache care and care received at the VHA. The survey is available in Supplementary Appendix A.

### Headache Impact Test (HIT-6)

Consists of six items that measure the impact an individual's headache has on their social functioning, role functioning, headache pain, vitality, cognitive functioning and psychological distress (16). Each item is rated (Never, Rarely, Sometimes, Very Often, Always) and coded into a numeric scale and summed to generate a total score ranging from 36–78. Higher scores indicate greater headache-related impact. Internal consistency is high (Cronbach's α = 0.89–0.90), and test-retest reliability is good, with internal intraclass correlation coefficient of 0.78 in general samples and 0.80 in stable headache groups (16).

### Pain, Enjoyment of life, and General activity (PEG-3)

Consists of three items that measure pain intensity and functional interference (21). Each item is rated using a 10-point Likert scale (0 = Does not interfere; 10 = Completely interferes) and the mean is calculated, with a higher score representing worse pain and interference. The PEG-3 shows good internal consistency (Cronbach's α = 0.73–0.89) and strong construct validity through correlations with established pain-specific measures (r = 0.60–0.95), and responsiveness to clinical change over time. Overall, the PEG-3 is a reliable and valid measure for assessing pain intensity and pain-related interference in patients with chronic pain (21).

### Pain Self-Efficacy Questionnaire (PSEQ-2)

A 2-item questionnaire that measures an individual's pain self-efficacy (22). The PSEQ-2 is a short-form version of the PSEQ, intended for screening purposes and smaller administrative burden. Items are rated on a 7-point Likert scale (0 = Not at all confident; 6 = Completely confident) and added together to get a range of 0–12. The higher scores indicate greater pain self-efficacy. The abbreviated scale has demonstrated excellent internal consistency (Cronbach α = 0.91) and good construct validity with strong correlations with the original PSEQ (r = 0.90) and evidence of convergent validity with measures of upper-extremity disability (QuickDASH; r = −0.61). Test-retest reliability was moderate (intraclass correlation = 0.66) (22).

### Insomnia Severity Index (ISI)

A brief, self-administered 7 item questionnaire that captures the type, intensity, and functional impact of adult insomnia symptoms. Items are rated on a 5-point Likert scale (0 = No problem; 4 = Very severe problem) and summed for a total score of 0–28, with a higher score reflecting greater insomnia problems (17). Internal consistency is high in both community (Cronbach α = 0.90) and clinical samples (Cronbach α = 0.91), convergent validity through significant correlations with measures of fatigue, quality of life, anxiety, and depression. There was strong criterion validity for identifying insomnia cases, and responsiveness to treatment-related changes in insomnia severity (17).

### Patient Health Questionnaire (PHQ-2)

A 2-item questionnaire that measures severity of depression. Items are rated on a four-point scale (0 = Not at all; 3 = Nearly every day) and based on the patient's experience within the last 2 weeks (18). The responses are summed for a total score of 0–6, with a higher score representing greater severity of depression. The PHQ-2 demonstrated good diagnostic performance for major depressive disorder, with 82.9% sensitivity and 90.0% specificity at a cutoff score of ≥ 3 (18).

### Generalized Anxiety Disorder (GAD-2)

A 2-item questionnaire that measures severity of anxiety symptoms. Items are rated on a 4-point Likert scale (0 = Not at all; 3 = Nearly every day) and are based on the individual's experiences in the last 2 weeks (19). The scores are summed and range from 0–6, with a higher score associated with greater severity of anxiety symptoms. At the recommended cutoff score of ≥3, the GAD-2 has demonstrated 86% sensitivity and 83% specificity for generalized anxiety disorder (19).

### ACORN (Social Determinants of Health)

The ACORN is a measure of 9 SDOH in the VHA (23). It had been iteratively developed and adapted based on feedback from VHA stakeholders including Veteran patients, and pilot tested in VA clinical settings (23). The determinants were selected by stakeholders based on ability to be addressed by VA resources, and included: housing instability, food insecurity, utility needs, lack of transportation, social isolation/loneliness, interpersonal violence, legal assistance, educational needs and employment concerns. Response items ask about current risk and include “yes/no”; “often true, sometimes true, and never true”; and “often, sometimes, never, not applicable” (23).

### Satisfaction with headache care and VA healthcare

We asked two single item 5-point Likert questions about Veteran satisfaction with their headache care received in the VHA and overall satisfaction with VHA healthcare (1 = Not at all satisfied, 5 = Very satisfied).

### Semi-structured interviews

We created an interview guide that expanded upon the Veterans' experience with headache prevalence, symptomology, and health care and toxic exposures during pre, active, and post deployment. Topics included the following: a. Burn pit and other toxic exposures during military service; b. History of headache and headache care; c. Headache Condition and Lifestyle Impact; d. Social Determinants of Health; and e. Recommendations for other Veterans and VA providers of headache care. Veteran patients were interviewed over Microsoft Teams software about their experiences across settings in the DoD and VHA, and SDOH. Several experienced team members conducted the interviews (TD, RB, SB, NR, LB, KS). We obtained recorded verbal consent for participation and virtual recording the interview. Interviews were recorded and transcribed using automated transcription within Microsoft teams. Transcripts were reviewed and cleaned for accuracy and de-identified prior to analyses. The interview guide is available in Supplementary Appendix B.

### Mixed methods analysis

We employed a mixed-method approach, using quantitative methods to address survey-related research questions and qualitative analyses to address interview-related research questions. Subsequently, qualitative data was explored stratifying by qualitative data.

### Quantitative analyses

Descriptive statistics were used to summarize key characteristics, symptoms, and outcomes of the study sample survey data. Data were inspected visually and tested for assumptions of normality using the Shapiro-Wilk test. Homogeneity of variances was evaluated using Levene's test when assumptions of normality were met and Brown-Forsythe test when normality assumptions were violated. When assumptions were violated, the Kruskal-Wallis test was used as the nonparametric analyses. Nonparametric analyses did not meaningfully alter the interpretation of findings, and as such parametric analyses are reported throughout for consistency. Independent *t*-tests and Analysis of Variances (ANOVAs) were used to compare group differences, with *post-hoc* comparisons and adjustments for type-1 error (e.g., Tukey's Kramer Honestly Significant Difference [HSD]). All analyses were conducted using Statistical Analysis System (SAS) Software, version 9.4 [(24)]. All analyses considered 2-tailed *p* < 0.05 as statistically significant. To assess the potential effect of missing or incomplete exposure data, we conducted a best/worst cast sensitivity analysis among those with completed outcome data. For individuals with missing exposure status, outcome analyses were rerun with individuals (1) assigned to the exposed group and (2) assigned to the unexposed group, and a summary comparison of the direction of findings is provided.

### Qualitative analysis

We used a Rapid Qualitative Analysis (RQA) approach using a standardized note template designed *a priori* to extract key concepts (Headache prior to deployment; headache during deployments; headache post deployment; headache self and health care across time and systems; headache phenotypes and headache symptoms and healthcare across deployment and systems) from each interview transcript and accompanying interview notes (25). Notes were reviewed, edited, and then entered an Excel matrix for analysis by the entire analysis team of 6 coders (TD, SB, KS, NR, RG, LB). All interviewers met initially to review and code several interviews to calibrate the coding and rapid data extraction for the matrix. After discussion, several adjustments were made to the RQA including multiple deployments across time; clarification of near open burn pits but no open burn pit duties; and specification of healthcare received in the community and VHA. This coding team met continuously to review the use of the template, make adjustments, and review key themes and quotes. If one team member disputed the thematic data, we discussed until we reached team consensus. For example, yoga was mentioned as self care; however VHA often offers yoga in the medical facilities. We decided to code as self care as noted in the interview context. All coders reviewed the RQA Matrix and had access to thematic analysis and had opportunities to dispute and offer additional analyses. We conducted case comparisons across the deployment periods of pre-deployment, deployment with DoD healthcare, and post-deployment with healthcare in the VHA. We conducted mixed-methods specifically using the headache phenotypes from the quantitative analyses to make qualitative comparisons of healthcare trajectories.

## Results

## Survey participants

Of the 1,980 eligible Veterans contacted, 772 (39.0%) responded to the survey. Among those, 338 did not report headache symptoms (10 denied headache, 328 did not complete the headache section of the survey), 99 respondents were screened out for potential secondary headache to PTH, resulting in a final analytic sample of 335 participants (16.9% of initial sample) see Figure 2. Survey respondents were majority male (*n* = 280, 83.6%), not Hispanic or Latino (*n* = 241, 71.9%), and White (*n* = 241, 71.9%). See Table 1 for full demographic information. The mean age at headache onset was 31.9 (standard deviation [SD] = 10.2) years.

**Figure 2:**
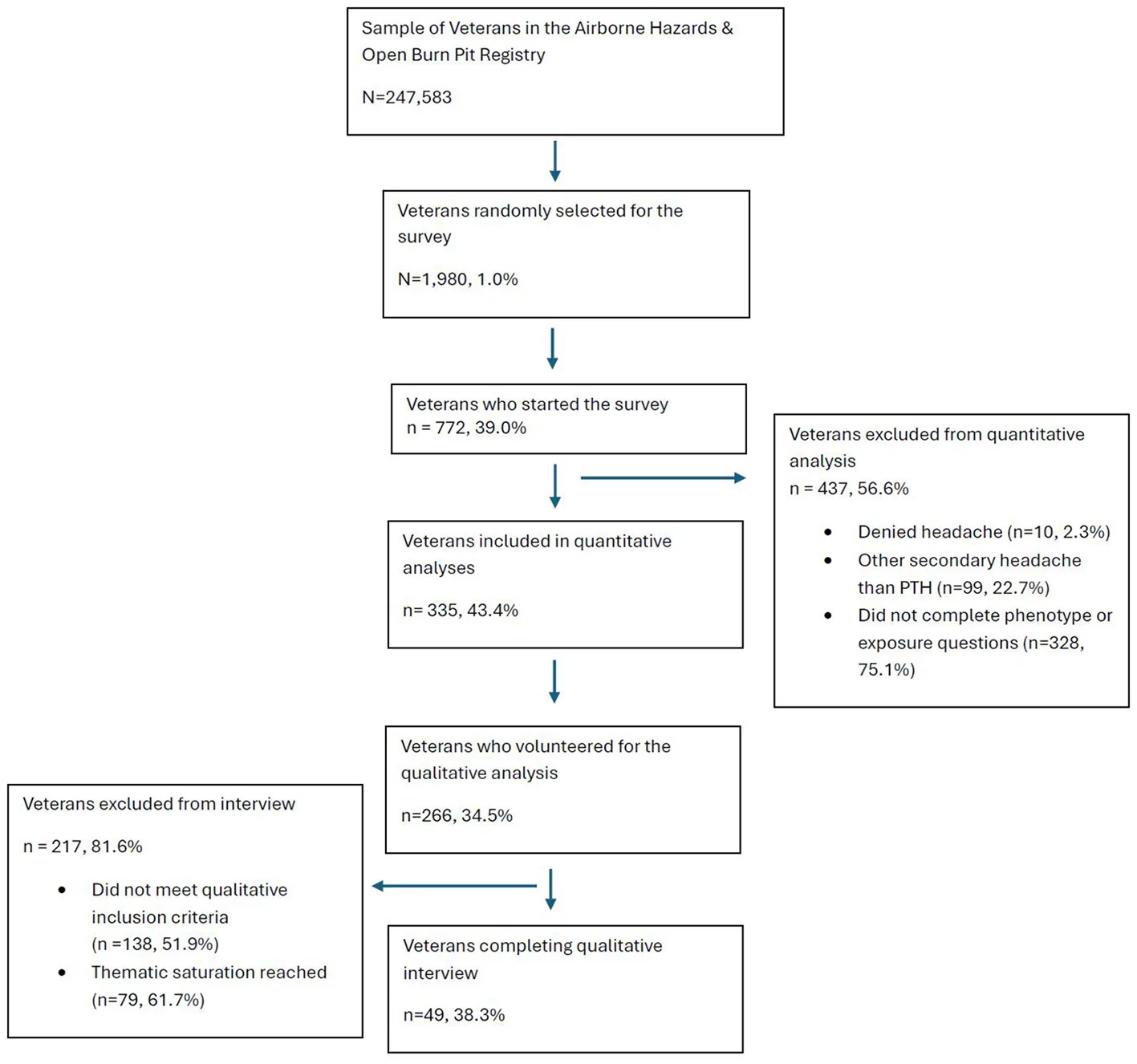
Recruitment process.

**Table 1:** Survey and interview participant demographics.

Characteristic	Headache phenotype participants (*N* = 335) (%)	Interview participants (*N* = 49) (%)
Sex		
Men	280 (83.6%)	32 (65.3%)
Women	51 (15.2%)	16 (32.7%)
No Response Given	4 (1.2%)	1 (2.0%)
Ethnicity (*n*)		
Hispanic or Latino	37 (11.0%)	5 (10.2%)
Not Hispanic or Latino	241 (71.9%)	34 (69.4%)
Unknown	4 (1.2%)	1 (2.0%)
Choose not to disclose	7 (2.1%)	1 (2.0%)
No Response	46 (13.7%)	8 (16.3%)
Race (*n*)		
American Indian or Alaska Native	9 (2.7%)	2 (4.1%)
Asian	15 (4.5%)	1 (2.0%)
Black or African American	47 (14.0%)	8 (16.3%)
Native Hawaiian	7 (2.1%)	0 (0.0%)
White	241 (71.9%)	34 (69.4%)
No Response	8 (2.4%)	2 (4.1%)
Unknown	2 (0.6%)	0 (0.0%)
Choose not to disclose	6 (1.8%)	2 (4.1%)
Total Years of Military Service (*n*)		
< 5 total years of military service	23 (6.9%)	4 (8.2%)
5–10 total years of military service	65 (19.4%)	14 (28.6%)
11–15 total years of military service	38 (11.3%)	3 (6.1%)
16–20 total years of military service	57 (17.0%)	6 (12.2%)
21–25 total years of military service	72 (21.5%)	10 (20.4%)
>25 total years of military service	74 (22.1%)	11 (22.4%)
No Response Given	6 (1.8%)	1 (2.0%)
Endorsed Traumatic Brain Injury (*n*)		
Yes	119 (35.5%)	17 (34.7%)
No	216 (64.5%)	32 (65.3%)

## Interview participants

Among those who completed the survey, 266 volunteered to participate in a follow-up interview, 128 were eligible, and 49 interviews were completed (Figure 2). Similar to the survey sample, the majority of the subsample of participants interviewed were male (*n* = 32, 65.3%), not Hispanic or Latino (*n* = 34, 69.4%), and White (*n* = 34, 69.4%) (See Table 1).

## Headache phenotypes among survey participants

Among the 335 individuals who completed the survey and reported headache, 311 endorsed open burn pit exposure. PTH was the most common phenotype among these individuals (*n* = 112, 36.0%), followed by migraine (*n* = 109, 35.0%), “other headache” (*n* = 78, 25.1%), and cluster headache (*n* = 12, 3.9%). Among the 112 with PTH, migraine was the most common phenotype (*n* = 75, 67.0%), followed by “other headache” (*n* = 24, 21.4%), and cluster headache (*n* = 13, 11.6%) (See Table 2).

**Table 2:** Headache phenotypes among veterans with and without open burn pit exposure.

Headache	With burn pit exposure (*N* = 311), *n* (%)	Without burn pit exposure (*N* = 24), *n* (%)
Post-traumatic headache	112 (36.0%)	7 (29.2%)
Migraine phenotype	75 (67.0%)	6 (85.7%)
“Other headache” phenotype	24 (21.4%)	1 (14.3%)
Cluster headache phenotype	13 (11.6%)	0 (0.0%)
Migraine	109 (35.0%)	8 (33.3%)
“Other headache”	78 (25.1%)	9 (37.5%)
Cluster headache	12 (3.9%)	0 (0.0%)

Those reporting headache without open burn pit exposure (*N* = 24) endorsed relatively higher rates of “other headache” (*n* = 9, 37.5%), and similar rates of migraine (*n* = 8, 33.3%), and relatively lower rates of PTH (*n* = 7, 29.2%). No cluster was endorsed in the non-open burn pit group. Among those with PTH, migraine was the most common phenotype (*n* = 6, 85.7%) (See Table 2).

## Headache phenotypes among interview participants

Within the subset of those who participated in a qualitative interview (*N* = 49), 12 were removed for potential secondary headaches to PTH. Among the remaining 37, PTH was the most common phenotype among these individuals (*n* = 17, 45.9%) followed by migraine (*n* = 11, 29.7%), “other headache” (*n* = 8, 21.6%), and cluster (*n* = 1, 2.7%). Among the 17 with PTH, migraine was the most common phenotype (*n* = 10, 58.8%), followed by cluster (*n* = 4, 23.5%), then “other headache” (*n* = 3, 17.6%) (See Figure 3).

**Figure 3:**
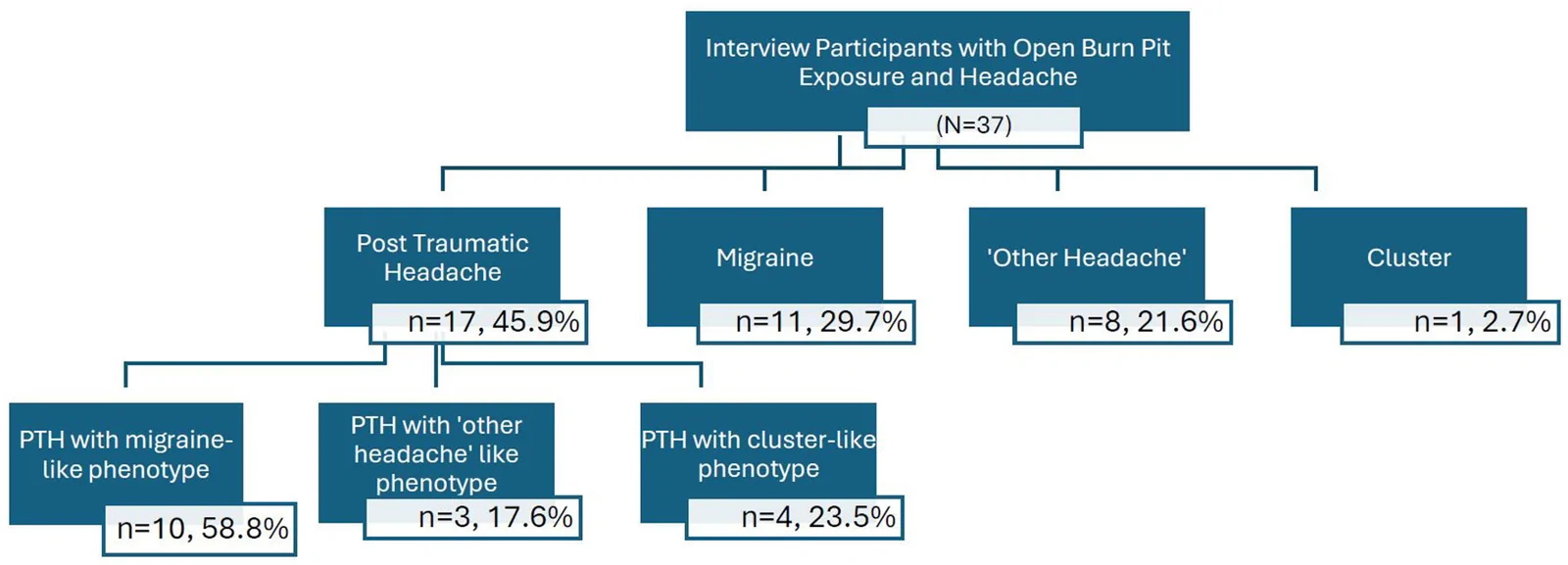
Interview participants with open burn pit exposure and headache.

## Headache and health outcomes between Veterans with and without open burn pit exposure

Survey respondents with headache and open burn pit exposure (*n* = 311) were compared to those with headache and no open burn pit exposure (*n* = 24) on several measures (Table 3). Those with open burn pit exposure reported significantly worse headache disability on the HIT-6 than those without open burn pit exposure (M = 61.47, SD = 7.61 vs. M = 58.21, SD = 8.93, *p* = 0.047, and 95% CI [0.04, 6.48]). Similarly, those with open burn pit exposure reported worse symptoms of insomnia on the ISI than those without open burn pit exposure (M = 15.47, SD = 5.90 vs. M = 11.88, SD = 5.28, *p* = 0.004, 95% CI [1.15, 6.04]). Other measures did not significantly differ between groups. Veterans with and without open burn pit exposure had similar mean ages at headache onset (M = 32.0, SD = 10.2 vs. M = 30.6, SD = 10.1 years, *p* = 0.537). Additionally, findings from the sensitivity analyses including 38 individuals with missing exposure data were generally consistent with the primary findings. The ISI remained significant in both analyses; HIT-6, PEG, PHQ-2, and GAD-2 varied by classification of unknown exposure, see Supplementary material S1.

**Table 3:** Headache outcomes by reported open burn pit exposure.

Outcome Measures	With open burn pit exposure and headache		Without open burn pit exposure and headache		*P*-Values	Mean difference	95% CI
	*n*	Mean (SD)	*n*	Mean (SD)			
Headache Impact Test	298	61.47 (7.61)	24	58.21 (8.93)	0.047	3.26	[0.04, 6.48]
Pain, Enjoyment of life, and General activity	305	5.08 (2.31)	24	4.11 (2.66)	0.051	0.97	[−0.01, 1.94]
Pain Self-Efficacy Questionnaire	294	7.99 (2.96)	24	8.33 (3.33)	0.589	−0.34	[−1.60, 0.91]
Insomnia Severity Index	308	15.47 (5.90)	24	11.88 (5.28)	0.004	3.60	[1.15, 6.04]
Patient Health Questionnaire	302	2.28 (1.82)	24	1.83 (1.93)	0.246	0.45	[−0.31, 1.22]
Generalized Anxiety Disorder	302	2.60 (1.96)	24	1.88 (1.78)	0.080	0.72	[−0.09, 1.54]
Frequency	274	47.84 (133.30)	21	31.02 (55.10)	0.252	16.82	[−12.40, 46.04]

## Headache and health outcomes between headache phenotypes

Additional group comparisons of survey respondents were conducted to determine differences across headache types among those with open burn pit exposure. Significant differences between headache types were observed across all measures (ps < 0.001 to 0.028). *Post-hoc* analyses demonstrated that significant differences were primarily driven by comparisons between PTH and “other headache” and between migraine and “other headache” across multiple outcomes (ps < 0.001 to 0.015). Exceptions include differences between migraine and “other headache” on the pain intensity and interference (PEG-2; *p* = 0.230) and depression (PHQ-2; *p* = 0.140), and between PTH and “other headache” on pain self-efficacy (PSEQ; *p* = 0.108). Additionally, cluster headache differed significantly from “other headache” for headache frequency (*p* = 0.004; see Supplementary material S2).

## Social determinants of health

There were 297 Veterans with open burn pit exposure who had a headache diagnosis and who completed the ACORN assessment. Of these, social isolation was the most commonly reported unmet need (*n* = 202, 68.0%). This was followed by a sixth of the sample reporting food insecurity (*n* = 45, 15.2%) or household utility concerns (*n* = 48, 16.2%). Other social needs included legal issues (*n* = 27, 9.1%), housing (*n* = 18, 6.1%), transportation (*n* = 17, 5.7%), and digital needs (*n* = 15, 5.1%).

## Qualitative findings about headache symptoms and healthcare by headache phenotypes

### PTH and “other headaches”

Many interviewees with the phenotype of PTH and ‘other headache' noticed increased frequencies of headache with military deployments. As one Veteran described

“[during] *various durations of military service I had ever increasingly more headaches*...” (#21049).

Many Veterans reported that their headaches started near the end of their deployment or after multiple deployments, suggesting that the headaches manifested after deployment to US military operations in the Middle East and Southeast Asia.

For example:

“*I started getting headaches after my third or fourth deployment and I couldn't figure out what was happening*.” (#22038)

Symptoms described were allergies, vision changes, breathing issues, TBI elevated blood pressure, and post-traumatic stress disorder (PTSD).

“*My vision changed and then I started getting sick and I started getting headaches... I have adjustment disorder, TBI, sleep apnea, and some serious muscular skin issues*...” (#22038)

“...*I myself was having breathing problems over there [deployment] and since I have been home*.” (#21049)

Veterans reported receiving both primary and specialty care in the VA and additional care in the community among those reporting both PTH and “other headaches”.

### PTH and migraine

Similarly, some Veterans mentioned experiencing more severe headaches during deployment, burning eyes and respiratory distress compared to no symptoms or headaches prior to deployment.

“*I have not had headache since before I was deployed... I have a headache every day since I have been back from deployment. I get very bad migraines that make me vomit*.” (#23266)

In addition, the Veterans reported experiencing chronic sinusitis and tinnitus, early stages of Chronic ObstructivePulmonary Disease (COPD) and headaches “that never go away.” (#12093).

“...*I came home in 2011 I have serious breathing problems...early stages COPD and these massive headaches*.” (#21037)

Multiple Veterans reported that during post-deployment they experience migraines very frequently and severely limiting their daily activities and work functioning because the migraines are accompanied by nausea and light sensitivity.

“*I am limited. If I do wake up with a headache that day, I am limited to pretty much nothing is going to happen that day. If anything, if I had anything planned because I can't do noise or light or anything of that nature*.” (#12093).

The majority of those with PTH and migraine reported receipt of VA primary and specialty care. Some choose to receive primary care in the community.

### PTH and cluster headache

Participants with this phenotype also reported severe headache symptoms post-deployment.

“…*When I got out (military), I just started getting these headaches and I actually thought I had like a brain tumor*…” (#22045)

These Veterans developed additional comorbidities including sleep apnea, vesicular migraines, auto-immune type problems as well as TBI and tinnitus following deployment.

“...*after my second deployment I developed sleep apnea within two years, and you know that was followed by vesicular migraines...I had vertigo*...” (#13036).

Most of the Veterans with this phenotype of PTH and cluster headache have been seen in the emergency room (ER) for the symptoms and care. Most received VA care including primary and specialty care (neurology).

### No PTH, cluster

Veterans with this phenotype have also reported severe comorbidities of sleep disorder, cancer and headache that began during active military service.

“*Shortly after working in the burn pit, I started developing the sleep disorder that I have, which was idiopathic hypersomnia...during my third duty station that's when the headaches started*.” (#22063).

These Veterans also reported receipt of ER care and were dual users of VA and Community care.

### No PTH, “other headache”

Veterans with this phenotype reported symptoms while on deployment including sleep apnea, chronic cough, elevated blood pressure and migraines on occasion.

“...*after that I developed severe allergies cause before that I didn't really have any problems with breathing or sinus issues...they were getting worse...I really couldn't breathe and even in the wintertime it was really just a prolong thing and then the constant headache go along with it*.” (#22205)

They reported some headaches continuing upon post-deployment but not as severe as the other phenotypes

“...*headache may last only a few seconds to a few hours*.” (#21020).

Majority of these Veterans report receiving community care with minimal VHA engagement.

### No PTH, migraine

Similarly, the Veterans reported that their migraines began during deployment and have worsened since then or started after their military service. Veterans noted prior to deployment, they had never experienced migraines.

“*My headaches and migraine started during the deployment...(migraine) has gotten worse, I get at least one or two a week*...” (#12037)

“...*it wasn't something I experienced until I went to Iraq*.” (#11015)

Post-deployment headache care varied widely across VA and in the community. Most report receiving VA care and one Veteran received headache medication from the VA but had chosen not to discuss headaches with VA primary care. A few Veterans were seen in the community for their healthcare. Only one Veteran received multidisciplinary VA care including pain management.

## Stepped headache care trajectories

We analyzed headache symptoms and Stepped-Headache Care (SHC) over their trajectories ranging from pre-deployment while in the DoD, US military deployment, post-deployment within the VHA and community care trajectories (See Table 4 for Stepped Headache Care Trajectories illustrations). Headache diagnoses reported by Veterans included migraines, general headache not specified, cluster headache, and TBI headache.

**Table 4:** Trajectories of Veteran headache burden and medical care journeys.

Veteran identifier	Pre-military deployment	Active duty deployment	Post-deployment	Stepped headache care
Veteran 1	No headaches prior to military deployment	Multiple Deployments over 24 years of active US military service. Began migraine medications during deployment. “*We were downwind from a burn pit area to where it smelled burned all the time.”*	Migraines continued post-deployment. Received Neurology Specialty Care in the Community for migraines and continued taking daily medications for migraines, has ALS. “*And that is one of the medications that I know I have to take every day, because if I miss it, those migraines will come right back.”*	Step 1 routine headache management during deployment. Step 2 Specialty Care Services post-deployment
Veteran 2	Headaches began during National Guard duty before Deployment. “*So technically, .. I started having issues [Headache] I was still in a drilling status.”*	Did not recall experiencing headaches during deployment; however headache intensity increased post-deployment. “*When I came back from my first one [deployment], I think it was like a couple years after I started experiencing these really, like intense, one sided headaches that wouldn't go over to like the other side.”*	Visited ER & Referred to Neurology in VA. Received prescription medications. “*[Headaches} wouldn't go away regardless of what I took until I actually went to the VA and I went to the ER at first and they said I had pink eye. I'm like, this is not pink eye. And they ended up giving me a neurology console.. And I talked to the neurologist about my symptoms, and they were still ongoing at the time too.”*	Step 1 and Step 2 in VA healthcare services post-deployment
Veteran 3	No headaches prior to military deployment, “*I have not had headaches before I deployed.”*	Direct Burn Pit Duties, No respirators, Used t-shirts around nose & mouth during deployment. “*Obviously there was a safety brief. Don't burn yourself, you know. But there wasn't any, … doing this could cause you X in the future. ”*	Visited ER and primary care; received prescription refills by mail “*I have a headache every day since I've been back from deployment…I get very bad migraines that make me vomit…I‘ve limited a lot of my activities. Based on just not wanting to get a migraine. It's very debilitating.”*	Step 1 routine headache care post-deployment
Veteran 4 (#11079)	No headaches prior to military deployment	Headache began in deployment, Also experienced TBI. Took OTC medications. No tests available in field during deployment.	Tried acupuncture out in the community on her own; used OTC medications. Reported headache “dulls but never goes away.” Received VA TBI care and VA primary care.	Foundational self-care during deployment and post-deployment. Step 1 VA routine headache care and Step 2 specialty care services.
Veteran 6 (#31125)	No mention of headaches prior to deployment	Self-medicated during active duty when headaches increased. DoD provided ibuprofen and provided diagnosis of “regular headache and sinus headache.”	Post-deployment and in retirement received VA healthcare where diagnosed with chronic migraine. Trialed many different headache treatments and medications and has seen many different specialists. Currently sees a neurosurgeon in the community and primary care in the VA. Practices yoga. Recommends for Veterans to ask more questions and ask to see a specialist.	Foundation Self-Care and Step 1 routine headache care in active duty and VA. Received Step 2 in the Community post-deployment.
Veteran 7 (#32077)	Experienced Migraines prior to deployment	Migraines progressively became worse during deployment with temporary blindness for several days.	Experienced migraines weekly. Received VA primary and specialty care services including neurology. Received advanced migraine treatments from VA neurology services.	Received Step 1 routine headache care and Step 2 Specialty Neurology Care in Post-deployment in VA healthcare.

## Self-care

During active-duty deployment, many reported they engaged in headache self-care with over the counter (OTC) medications. Post deployment, concurrent to receiving primary and/or specialty healthcare, some Veterans reported trying self-care strategies to manage their headaches, including acupuncture and yoga outside of the healthcare system. However, all who tried these modalities noted they did not help with their headache symptoms (See Table 4).

Some Veterans reported they were unaware of treatment options available for headache post-deployment and recommended that other Veteran patients ask their healthcare providers about all the available treatment options and possible referrals to specialists to find optimal headache treatment plans.

## Headache care pre-military deployment

A handful of the interview participants noted having experienced either “normal headaches” or migraines before any military deployments that were minor in terms of their symptoms and impact. However, the majority reported no headaches experienced prior to military operations deployment in the Middle East and/or Southwest Asia.

## Headache care during military deployment

Most of the interview participants described experiencing their first-ever headache during military deployment or soon after return, which included exposure to open burn pit smoke and fumes either directly through duties or indirectly through living nearby.

Self-care (foundation level SHC) was the most frequently utilized headache management approach during deployment. Veterans indicated that often there were no medical testing machines or prescription strength medications/devices available during field operations when deployed compared to stateside care. Most reported taking OTC medications (e.g., ibuprofen). Some reported that they rested during headache episodes, while others reported remaining on duty. One participant reported that most service members in their unit they were deployed with were unfamiliar with headache diseases. No interviewees reported discussions about receipt of preventive headache care during deployment (see Table 4).

## Headache care post-military deployment

Most Veterans explained that headache symptoms and poor headache functioning either began or progressively worsened post-deployment. Participants with continuous headache post-military deployment also reported experiencing continuous headache symptoms.

Others described how debilitating their headaches remain post-deployment, impacting their daily living activities (See Table 4).

## Post- deployment healthcare

Headache care often stepped up from foundational self-care to routine headache care in primary care settings, (Step 1) and eventually progressed to Step 2 with specialty care services providing advanced headache care during post-deployment.

“*The treatment [*for headache*] initially started on active duty and when I got out. As when I started getting more intense, I don't want to say intense treatment, but I started getting better medications*.” (#32077)

Most of the Veterans were receiving VHA healthcare or were dual users of VHA and community care. Their point of entry into the healthcare system varied and often included the ER with referral to neurology, and then back to primary care for medication management and refills. Referral to the TBI clinic was also a point of entry into VHA with return to primary care after stabilization. Some Veterans reported that they were prescribed OTC medications or other headache treatments in primary care (Step 1) while others received Step 2 Specialty care services (e.g., neurology) in the VHA and in the community, including botulinum toxin injections (e.g., onabotulinumtoxinA), other injections, nerve blocks, and trials of headache medications and treatments.

One Veteran reported being diagnosed with a headache disorder while in active duty and receiving medications in the VA post-deployment to manage the headaches.

“*I was diagnosed with headache during active duty. My primary care provider (VHA) makes sure that I have the medicine that I need available to m*e [to manage headaches].” (#12093).

Some Veterans reporting receipt of headache care out in the community by primary care, including headache-specific treatment.

## Discussion

This mixed-methods study provides the first phenotypical characterization of headache among Veterans with self-reported open burn pit exposure and developed a patient-centered understanding of what that headache disease looks like clinically, how it impacts Veterans across multiple headache and headache-aligned functional domains, and describes headache care for Veterans from the deployment field through their transition into the VHA. A plurality of Veterans endorsed PTH with migraine as the dominant phenotype. Of those meeting criteria for primary headache, migraine was the most frequent. Veterans with open burn pit exposure reported significantly greater headache disability and insomnia severity compared to Veterans with headache but without exposure. As expected, those with, migraine and PTH consistently reported greater burden than those “other headache”.

Among those exposed to burn pit, headache usually began into their thirties, a time in which most Veterans have left military service. Qualitative interviews contextualized these findings by revealing that headache was largely absent prior to deployment, developed or worsened in direct temporal relationship with burn pit exposure, and progressively chronified post-deployment. Furthermore, headaches were often accompanied by respiratory, vestibular, and neuropsychiatric comorbidities that compounded the overall disease burden. Together, these findings carry direct implications for clinical phenotyping of PTH and broader headache care delivery, social determinants screening, and health policy for the growing population of post-9/11 Veterans affected by toxic airborne hazard exposures.

To our knowledge, this study offers the first systematic phenotypical description of headache in an open burn-pit-exposed Veteran cohort using ICHD-3 criteria and AMPP-derived classification methodology (12, 13). PTH was the most prevalent phenotype (36.0%), followed by migraine (35.0%) “other headache” (25.1%), and cluster headache (3.9%). A clinically critical finding was the internal composition of the PTH group: two-thirds of Veterans with PTH were further classified with a migraine sub-phenotype (67.0%). Recent literature supports a primarily migraine-like phenotype among those with PTH (26). Across outcomes, PTH appeared functionally similar to migraine in this sample, though other studies suggest PTH is accompanied by a higher disease burden, including more persistent or longer headaches. The biological plausibility of a preferentially migrainous response to burn pit toxicants is well-supported by the environmental health literature. Open burn pits incinerated plastics, rubber, chemical waste, munitions, and human waste using JP-8 jet fuel as an accelerant, releasing fine particulate matter, volatile organic compounds (VOCs) including benzene, and polycyclic aromatic hydrocarbons (PAHs) (27). These inhaled toxicants can penetrate the respiratory system, enter systemic circulation, and cross the blood-brain barrier, while also accessing the central nervous system (CNS) directly via the olfactory tract (28). Once in the CNS, these substances trigger the release of pro-inflammatory cytokines, including interleukin-1β (IL-1β) and tumor necrosis factor-alpha (TNF-α), leading to sustained neuroinflammation and oxidative stress, and producing chemical irritation of trigeminal sensory endings in the respiratory and sinonasal mucosa, a key pathway in the initiation and maintenance of migraine attacks (29). Veterans with chronic exposure exhibit higher rates of neuroinflammatory markers and accelerated cognitive decline, mirroring mechanisms observed in populations with sustained urban air pollution exposure (28). These mechanistic pathways provide a compelling explanation for why those with open burn pit exposure would more frequently manifest migraine phenotypes rather than non-specific or “other headache”. Clinically, this implies that Veterans presenting with PTH following deployment with open burn pit exposure may benefit from phenotype-guided management; for those with a migraine phenotype, evidence based migraine specific treatments should be considered, including consideration of calcitonin gene-related peptide [CGRP]-targeted therapies and preventive treatment (30). Notably, cluster headache was absent among the 24 Veterans without burn pit exposure, a finding likely reflecting the low sample size and relative rarity of cluster diagnoses (31). However, this may represent an additional exposure-specific phenotypic signal, requiring replication in larger samples.

The integration of validated patient-reported outcome (PRO) measures in this study demonstrates how open burn-pit-related headache is associated with substantial burden on functioning, sleep, mood, and pain. Among this headache sample, those with open burn pit exposure reported worse headache disability and symptoms of insomnia compared to those without open burn pit exposure. Although differences were not observed between other outcomes, including emotional, self-efficacy, and pain and pain interference, this is most parsimoniously explained by the small non-exposed comparator group (*n* = 24), which substantially limited statistical power. Notably, the directional trend across all measures favored worse outcomes in the exposed group. The PRO data also carry direct policy relevance: by showing that open burn pit exposure is associated with worse outcomes, this study provides initial evidentiary support for the classification of chronic headache as a presumptive condition under the PACT Act (2022), which expanded VA health care benefits to millions of post-9/11 Veterans (1).

Among the open burn-pit-exposed Veterans, phenotype-level comparisons revealed that migraine and PTH carried a consistently and significantly greater burden than “other headache” types across nearly every measured health domain. Notably, migraine and PTH did not significantly differ from each other on any measure. This functional equivalence between migraine and PTH is likely attributable in large part to the dominance of migraine sub-phenotype within the PTH group (67.0%). This study's finding that the burden of PTH is similar to that of primary migraine in an open burn pit exposed cohort adds a new dimension to the PTH literature and has direct implications for clinical guideline development. In the interim, approaching PTH treatment with a migraine-first framework appears indicated. Veterans with non-PTH “other headache” represented the lowest-burden subgroup and, consistent with the quantitative findings, were also the group least engaged with VHA specialty services in the qualitative data.

Consistent with emerging evidence that environmental toxicant exposure can sensitize central pain pathways over time (27), the qualitative findings reveal a coherent and phenotype-spanning pattern of headache onset or worsening during deployment followed by progressive chronification post-deployment. The majority of interview participants reported no headache prior to military deployment; new-onset headaches emerged during or shortly after deployment, often in close temporal relationship with open burn pit exposure, with Veterans describing headaches arising after their third or fourth deployment and worsening exponentially over successive tours. This temporal arc of exposure, new-onset or worsening headache, and post-deployment chronification with accumulating disability, is clinically significant and parallels findings in environmental health research on fine particle matter and headache, which demonstrate that chronic, cumulative inhalation of fine particulate matter produces dose-dependent neurological sensitization (28). The relationship identified previously, with Veterans in the highest cumulative open burn pit exposure quartile (>448 days) showing approximately 55% higher odds of migraine diagnosis, provides an epidemiological counterpart to the subjective escalation reported by Veterans in this study (6). Veterans across all phenotypes described post-deployment headache as more severe, more frequent, and more disabling than during deployment, with daily headaches, nausea, vomiting, photophobia, phonophobia, and loss of occupational and social functioning commonly described in the migraine and PTH phenotype groups. Comorbid respiratory disease (COPD, chronic sinusitis), vestibular dysfunction, tinnitus, sleep apnea, TBI, and PTSD were reported across phenotype groups, reflecting the polysystemic burden of sustained toxic inhalation exposure and is consistent with prior evidence that open burn pit exposure is associated with a broad range of chronic conditions (5). The convergence of qualitative narrative and quantitative data on this progression toward chronification represents one of the strongest demonstrations of methodological triangulation in this mixed-methods study.

The application of the VHA SCM-PM (7) as an evaluative framework for Veterans' headache care trajectories revealed persistent and structural gaps in guideline-concordant care across all phases of military service and post-deployment transition. The care trajectories documented in this study demonstrate that SCM principles are not being reliably operationalized for open burn pit exposed Veterans with headache. During deployment, care was constrained to foundational self-care (Step 0) by operational necessity. Over the counter (OTC) nonsteroidal anti-inflammatory drugs (NSAIDs; predominantly ibuprofen) were the universal abortive treatment. No prescription medications or diagnostic equipment were available in field environments and no preventive headache therapy was initiated. At least one Veteran reported that the deployed unit was entirely unfamiliar with headache disease.. This reliance on OTC NSAIDs, without access to first-line guideline-recommended abortive treatments such as triptans, or preventive therapies such as topiramate, propranolol, or amitriptyline, represents a critical missed window for early intervention during a period of peak and ongoing exposure (30). Service members themselves likely had less incentive to initiate higher levels of care given the lack of information available at the time of their deployments related to long-term neurological risks of open burn pit exposure. Indeed, Veterans in this study reported briefing focused entirely on the physical dangers of open burn pits.

Post-deployment care trajectories were wide-ranging and frequently fragmented care departing from the tiered model of the SC framework. Rather than progressing from primary care (Step 1) to specialty care (Step 2), Veterans often entered the healthcare system through ERs, sometimes repeatedly, before obtaining neurology referrals. Others were routed through TBI clinics, reflecting the high -occurrence PTH in this cohort. These trajectories mirror findings in prior research on post-deployment chronic pain, which documented that multiple “points of entry” into the VHA system, rather than structured progression through pain management (32). Veterans who ultimately reached Step 2, or specialty care, (e.g., neurology, TBI clinics) described receiving advanced treatments including botulinum toxin, nerve blocks, CGRP-targeted therapies, and neurotoxin injections; however, this progression typically required persistent self-advocacy. Veterans described needing to “ask a lot of questions” and repeatedly request specialist referrals. A subset of Veterans pursued community-based neurology (including neurosurgeons) alongside or instead of VHA care, citing perceived barriers or prolonged wait times, consistent with previously documented patterns of dual VA and community care use among Veterans in this population (33). Concurrent foundational self-care persisted across all post-deployment phases, including acupuncture and yoga. The PACT Act's expansion of VA access to open burn pit exposed Veterans is a critical policy advance; however, expanding access without simultaneously implementing structured care navigation and open burn pit exposure-aware stepped-care pathways risks perpetuating the fragmented, patient-driven trajectories documented in this study (34).

This study is, to the authors' knowledge, the first to apply a systematic SDOH screening tool, VHA's ACORN instrument (23), in the context of open burn pit associated headache, and the findings reveal a profound and actionable social burden that has not been previously characterized in this population. The finding that 68.0% of respondents (202 of 297) reported social isolation or loneliness exceeds estimates reported in broader U.S. Veteran populations (56.9%), suggesting a substantial burden in this cohort (35). Social isolation and loneliness are established, independent risk factors for early mortality (29% and 26% increased likelihood, respectively) (34) and for coronary heart disease and stroke (36). In the context of migraine and PTH, social isolation operates bidirectionally: it is both a consequence of headache disability, as Veterans withdraw from work, family, and leisure activities during headache episodes, and a potential exacerbating factor through its impact on mood dysregulation, pain catastrophizing, and disengagement from healthcare. The current Stepped Care Model (SCM) does not incorporate SDOH assessment as a structured, mandatory element across all tiers; the present findings argue that it must. Future research should examine whether SDOH burden mediates or moderates the relationship between headache phenotype severity and healthcare engagement, an important question that the cross-sectional survey design cannot fully address but that the ACORN findings strongly motivate.

The mixed-methods design of this study represents a methodological contribution that extends beyond the clinical findings themselves. The combination of RQA of 49 semi-structured interviews with quantitative analysis of 335 survey respondents drawn from a registry-linked parent sample of 247,583 Veterans achieves a degree of internal and external triangulation that neither method could accomplish alone. The RQA approach, employing standardized *a priori* templates reviewed iteratively by the full analysis team, is a methodologically appropriate choice for this clinical context and has been validated as capable of delivering rigorous findings within compressed timelines (25). The quantitative data establish preliminary evidence that open burn pit exposed Veterans have significantly worse headache disability than non-exposed counterparts. The qualitative data explain why through first-person narratives of inadequate field-care resources, delayed specialty access, systemic unfamiliarity with headache disease in deployment settings, and the persistent self-advocacy burden required to navigate post-deployment care. Together, they deliver what Creswell and Plano Clark describe as a “complete picture” of the total illness experience that transcends the limitations of either method alone (37). The directional but non-significant differences between exposure groups on other pain, mental health, and self-efficacy outcomes, juxtaposed against qualitative descriptions of profound psychological distress, underscore the value of mixed-methods triangulation. The qualitative data provided contextual depth to offset the likely underpowered non-exposed group and recognize that psychological burden is nonetheless real and clinically meaningful in this population.

Beyond psychological stress and other deployment-related factors, several potentially complementary pathways, including systemic and central inflammation, oxidative stress, trigeminovascular activation and sensitization for migraine, and sleep–circadian–hypothalamic dysregulation for cluster (6, 38). Experimental support for a lung–brain inflammatory pathway comes from a recent rodent model of military burn-pit exposure in which inhalation of carbon black and naphthalene, used as surrogates for particulate and volatile components of burn-pit emissions, produced inflammatory and metabolic alterations in both lung and brain tissue, including increased nuclear NF-κB signaling in the brain (39). Similar findings have been observed following inhalation of ambient particulate matter, which increased brain concentrations of the proinflammatory cytokines IL-1α and TNF-α and activation of NF-κB in experimental models, providing additional evidence that inhaled particulate exposures can promote neuroinflammation (40). These inflammatory and oxidative pathways could be particularly relevant to migraine because combustion-derived irritants such as acrolein can activate TRPA1-expressing trigeminal sensory afferents, resulting in CGRP-dependent neurogenic signaling and meningeal vasodilation. This may provide a potential interface between inhaled toxicants and the trigeminovascular system (41). Moreover, repeated exposure to environmental irritants has been shown experimentally to sensitize trigeminovascular responses, suggesting that recurrent or prolonged inhalational exposures might lower the threshold for headache generation in susceptible individuals rather than acting solely as an acute headache trigger (42).

Mechanisms relevant to cluster headache may additionally involve the hypothalamic and circadian systems, as functional neuroimaging has demonstrated hypothalamic activation during cluster headache attacks and clinical studies have demonstrated altered circadian secretion of melatonin and cortisol as well as pronounced circadian rhythmicity of attacks (43–45).

These findings carry direct and actionable implications for clinical practice, healthcare system design, and Veterans' health policy. First, exposure-aware headache assessment should be adopted as standard of care for post-9/11 Veterans. Screening for open burn pit exposure became mandated following the 2022 PACT Act, though no guidelines have been implemented for those endorsing exposure. Military and VA providers should routinely integrate an occupational and environmental exposure history, including duration, proximity, and duties related to open burn pits, into all headache assessments, mirroring the occupational medicine standard of exposure-first clinical inquiry (46). Veterans reporting proximity to open burn pits with direct waste-management duties, the highest-risk group in previous studies (6), may warrant particular attention during headache assessment and referral for specialty care if symptoms are refractory to initial Step 1 management.

Second, early preventive treatment must be available during deployment. The deployment phase represents a critical but currently missed window for preventive headache intervention, regardless of open burn pit exposure. Deployment formularies and medical readiness protocols should be reviewed to include first-line preventive agents (e.g., topiramate, propranolol, amitriptyline) and prescription abortive treatments (e.g., triptans) for service members with new-onset migraine or PTH during deployment, rather than defaulting exclusively to OTC NSAIDs (47).

Third, structured patient navigation services need to be improved to bridge the DoD-to-VHA transition. The fragmented, patient-driven care trajectories documented in this study provide direct evidence for the value of dedicated patient navigators within VA Headache Centers of Excellence (HCoE). Navigators should be trained in exposure history, SC escalation criteria, SDOH referral pathways, and should serve as a proactive interface between military transition and VHA enrollment.

Fourth, assessment of SDOH should be integrated as a parallel element of the SCM at every tier, given the high rates of social isolation household utility concerns. The VHA's existing ACORN infrastructure provides a ready platform for this integration (23).

Fifth, phenotype-guided management should be formalized for open burn pit exposed Veterans with PTH. Given the predominance of migraine phenotype within PTH in this cohort and the equivalent functional burden of PTH and migraine, clinical pathways should default to migraine-specific management, including CGRP-targeted therapies and neuromodulation devices (47, 48).

Sixth, regarding PACT Act and presumptive conditions policy: the evidence generated by this study, demonstrating significantly worse headache disability in exposed vs. non-exposed Veterans with headache, provides initial evidence for the formal inclusion of migraine and PTH as presumptive conditions for Veterans with documented open burn pit exposure under the PACT Act framework (1, 49).

## Limitations

Several limitations of this study warrant acknowledgment. First, this study is observational and does not establish causation; the retrospective and cross-sectional design precludes the temporal inferences that would be available from prospective longitudinal data. Additionally, data was not available to control for other potential deployment-related contributors to the observed findings, such as diagnosed chronic pain or mental health conditions. The absence of a baseline pre-deployment headache assessment for the survey participants limits the ability to definitively attribute new headache onset to deployment exposures, though qualitative interviews provide corroborating pre/during/post-deployment temporal data from participants themselves. The response rate of this survey, while high for this type of methodology (17%), may still result in a bias based on who is motivated to respond (e.g., higher disease burden, greater VHA engagement). These findings may not be generalizable to the broader population of open burn pit exposed Veterans. Information was not available for nonresponders; therefore, responder and nonresponder characteristics could not be formally compared. Although sensitivity analyses were conducted to assess the impact of unknown burn pit exposure status, not all participants with unknown exposure could be included due to missing The non-exposed comparison group was small (*n* = 24), substantially limiting the statistical power of between-group comparisons and likely explaining the directional but non-significant differences observed on pain, pain interference, mood, and self-efficacy. Exposure data relied on self-report from the AH&OBP Registry; no gold-standard environmental measure exists for determining the specific chemical magnitude of exposure for an individual service member, and the Healthy Warrior Effect, the selection of a generally healthier cohort into military service, may result in an underestimate of the true disease burden (49). Participants in this study were classified into a primary headache phenotype, even though headache disorders commonly overlap. Some participants with multiple co existing headache phenotypes may not have been fully represented, introducing the potential for phenotype misclassification. Finally, qualitative interviews relied on voluntary participation and may not capture the full diversity of Veteran experiences, particularly those with the most severe functional limitations who may face barriers to study participation. Despite these limitations, the mixed methods design enabled triangulation of quantitative and qualitative findings, substantially strengthening the internal validity of the conclusions and providing a more comprehensive understanding of how open burn pit exposure intersects with headache disease and healthcare utilization than either method alone could provide.

## Conclusion

Veterans exposed to open burn pits during military deployment represent a large, growing, and clinically underserved population for whom headache, predominantly migrainous in phenotype, constitutes a high-burden, chronic, and often inadequately treated disease. This study establishes that headaches among those with open burn pit exposure is not a homogeneous: phenotype matters clinically, with migraine and PTH representing a high-burden subgroups distinguished by equivalent and severe disability across functional, psychological, and sleep domains. The healthcare trajectories documented here reveal a system that is not yet meeting these Veterans where they are, neither during deployment, when prevention is most impactful, or during the post-deployment transition, when structured navigation and SDOH-integrated care could interrupt the chronification process. Building on previous epidemiological studies associating open burn pit with headache (6), these findings further support for a shift toward exposure-responsive clinical care, one that integrates phenotype-guided headache management, SC navigation, and social determinants screening into a cohesive, proactive treatment model for all Veterans enrolled in the AH&OBP Registry (6). Future research should prioritize longitudinal studies to evaluate whether early, guideline-concordant intervention during and immediately after deployment can prevent the chronification trajectory documented here and should investigate objective neuroinflammatory biomarkers to validate the mechanistic link between open burn pit toxicant exposure and trigeminovascular activation in this population (29).

## Data Availability

The raw data supporting the conclusions of this article will be made available by the authors, without undue reservation.
