# Supplementary material for: Veteran deployed burn pits exposure: headache phenotypes, trajectories, and outcomes: a mixed-methods evaluation
Source: Front Neurol. 2026 Oct 22;17:1899580. doi: 10.3389/fneur.2026.1899580 (PMC13735539; doi:10.3389/fneur.2026.1899580)
Supplement: Supplementary file 1 [file Data_Sheet_1.pdf]

Burn Pit Veteran Interview Guide

Participant ID:

Group:

Date

Prior to recording:
Hello, my name is [NAME] and I work with the VHA Headache Center of Excellence (HCoE) Research and Evaluation Center.

We are interviewing you today because, based on your survey response, you have been identified as someone with headache symptoms and/or a headache condition and may have had burn pit exposure during your military service.

Our study team is helping the HCoE improve experiences for Veterans receiving headache care. One way we are doing this is by talking to Veterans, like yourself, to find out more about their experiences. Participation in this study is voluntary; you are free to withdraw at any time. Data that are already collected prior to you withdrawing may be used in the study results, however, no new information will be collected or used. You have the right to withdraw permission for the use of your data in this research study at any time. We will keep what you tell us confidential within our team, and we will not link your name or any identifying information in reports of study findings to the HCoE, VHA, or elsewhere.

If you have any concerns or questions regarding the conduct of this project you may contact the project lead, Dr. Jason Sico at 203-648-3068.

Do you have any questions about our project?

To make sure we correctly document responses, we record our interviews. All interviews will be transcribed and de-identified to protect your identity, you will not be identified by name. Do you consent to this interview being recorded? Your response will not affect your ability to participate in the interview.

[If yes, begin recording and read recorded consent statement]
[If no, do not record but read the consent paragraph and then proceed with the interview, taking written notes of responses]

Once you have approval to record the interview, turn the recorder on and state:
"Today is (DATE) and this is participant (participant ID#). Thank you for your participation in this project. We appreciate your time. Today we are here to learn about your experience with headaches and/or your migraine disorders, and your experience with open burn pits and other toxic exposures. We are conducting these interviews to best inform the VA's new Headache Centers of Excellence initiative. We are very interested in your thoughts and opinions on what you want healthcare providers to know about your headache, and headache care. The information you share today may help improve care for Veterans seeking headache care in the future. Do you agree to be interviewed [and recorded]?"

[If yes to interview and recording, ask participant to state agreement and then proceed with interview questions]
[If no to recording but yes to interview, do not record but proceed with the interview, taking written notes of responses]
[If no to interview, thank the person and end the call.
Thank you for your service. The first set of questions asks about your military service.

Section A: Burn Pit & Other Toxic Exposures

1. What branch of the military were you in?
    a. What was your rank and Military Occupation Specialty (MOS)?
    b. What were your typical duties?
    c. How many deployments did you have?
        i. Where were you deployed (each time)?
        ii. For how long were you there (each time)?
2. [If not already mentioned] Do you think you were exposed to open burn pits while deployed?
    a. Please describe the circumstances. (probe for where, how direct, what length of time, etc.)
    b. What were normal procedures or activities that involved burn pits?
        i. Was your experience different from others in your unit? In what ways?
    c. At the time, do you recall anyone warning you about the exposure, or talking about it in any way?
        i. What did you hear about exposure?
    d. At the time, do you recall any concerns you had about this exposure?
        i. If yes, what were your concerns?
        ii. If yes, who did you talk with, if anyone, about your concerns?
        iii. Did you ever receive treatment related to exposure to burn pit while you were deployed?
            1 If yes, what led you to seek treatment?
            2 How did you feel about the process of asking for and getting treatment for exposure?
3. *Outside of open burn pit exposure*, did you experience any other military or environmental exposures? (If needed, for example chemical agents and explosions)

   a. <u>Potential exposures for Afghanistan</u>: explosion; firearm discharge noise; other loud noise; sand, dust and particulates; toxic embedded fragments; infectious disease; rabies; mefloquine to prevent malaria; heat exposure; cold exposure; depleted uranium; occupational hazards; chemical warfare agents
   b. <u>Above and additional for Iraq</u>: Sulfur fire; chromium
   c. <u>Above and additional for Gulf War</u>: vaccinations for anthrax and botulinum toxoid; oil well fires; CARC paint; PB tablet to protect against nerve agent soman; pesticides

4. In what ways, if any at all, do you think your headaches might be related to any of your past military exposures?

   a. Probe for any difference in, or onset of, headache symptoms during and/or after exposure.

5. Have you ever spoken to anyone – other Veterans, friends, family, health care providers – about your thoughts regarding potential impact of toxic exposures on your headache symptoms?

   a. If yes, Who did you talk with and did you first bring up the topic or the other person did?

      i. When did you talk with them?

      ii. What made you want to talk with them?

      iii. What did you talk about?

6. When you were exposed to [xxxxx], were you offered PPE?

   a. If yes, What were you told about the need to use PPE?

   b. What type of PPE was made available?

   c. Did you find that you were able to use the PPE regularly? If not – what got in the way?

      i. In what ways was PPE actually used?

   d. How adequate do you think the PPE was to protect you from the toxic or noise exposures?

7. *[If had burn pit exposure]:* Have you sought or received disability pay related to your burn pit exposure?

   a. If yes, Please tell me about that.

   b. Do you receive disability compensation related to burn put exposure?

Next we will shift our conversation to headaches for which you have been receiving VA care.

Section B: General Considerations regarding History of Headache and Headache Care

8. When did you first start experiencing headache? [before military service, during, exacerbated during military service, after discharge]

9. If before military service: What were the headache symptoms like?
10. If started during military service or became exacerbated during military service: What were the headache symptoms like?
    a. If you feel comfortable telling me, what do you think brought on your headaches?
    b. How severe were your headaches?
    c. How did your headache symptoms change after deployment/during active duty?
    d. How did you manage the headache symptoms?
    e. What prompted you to seek medical help for headache the first time?
        i. What got in the way, if anything, of you seeking treatment for headache while deployed? (probe for perception of headache severity; stigma; perceived access to headache care; etc.)
        ii. What helped you, if anything, get treatment for your headache while deployed?
    f. Did you receive any headache treatment when receiving military health care?
        i. If yes, Please tell me about your early experiences with headache treatment while deployed or during active duty.
11. If started after discharge: How soon after discharge did your headaches start?
    a. What were the headache symptoms like? How bad were your headaches?
    b. How did you manage the headache symptoms?
    c. What prompted you to seek medical help for headache the first time?
        i. Where did you seek treatment?
        ii. What got in the way, if anything, of you seeking treatment for headache? (probe for perception of headache severity; stigma; perceived access to headache care; etc.)
        iii. What helped you, if anything, get treatment for your headache?

12. *[If this does not come up in previous question, or is not fully covered in previous question]:* Please tell me about your experience receiving headache care at the VA. (Probe for over the years, Last visit, where, primary care provider or someone else, how satisfied were you with visit.)

13. *[If not fully covered already]:* What type of health care providers do you currently see at the VA specifically, for your headache? [Primary Care physician, Neurologist, Psychologist, Psychiatrist, a specialist you were referred to by your primary care provider; pharmacist; Other_________ [massage therapist, chiropractor, acupuncturist].

14. Comparing the headache care that you receive now compared to the headache care you first received when you started having headaches, what (if anything) is different? (probes: have medications changed, were you initially only given as needed medication, other types of therapies like relaxation)?

15. How well do you think your headaches are managed currently? What makes you say this?

16. *[If not addressed already]*: Have you ever had a clinical pharmacist as part of your health care team for headache care? Here I mean involved in your care beyond filling a medication prescription, and meeting with you to help manage your treatments.
    a. *[If yes],* How was the pharmacist involved?
        i. What did you think of having a pharmacist involved in your care?

ii. How satisfied were you with the pharmacist's care, on a scale from 1-5, where 1=completely dissatisfied, 3=neither satisfied or dissatisfied, and 5=completely satisfied?
iii. Why this rating?

17. *[If not addressed already]:* Have you had any rehab services for your headache care? By rehab services, I mean care from an occupational therapist (OT), and/or a physical therapist (PT), recreational therapist, physiatrist, other.
    a. *[if yes]* What kinds of services did you receive?
        i. How satisfied were you with those rehabilitation services on a scale from 1-5, where 1=completely dissatisfied, 3=neither satisfied or dissatisfied, and 5=completely satisfied? Why this rating?

18. Have you received any headache treatment in the community? Please tell me about that experience.

    [If not already addressed]: Now I'd like to ask you about your headache ***treatments*** *that you have received from any source of care over the years.*

19. What is important to you in the treatment plan for your headache and how do you discuss what is important to you with your healthcare providers?

20. Thinking back through all your years of receiving treatments for your headaches, what types of treatments have you tried and how have they worked for you?

21. Overall, thinking about all the treatments you have NOW, what do you consider to be the most helpful or most important aspect of your prescribed treatment for your headaches?

    To get an overall rating of your headache care and the providers delivering this care, now:

22. And how satisfied would you say you are with your VA headache care, overall?
    i. On a scale from 1-5, where 1=completely dissatisfied, 3=neither satisfied or dissatisfied, and 5=completely satisfied?
    ii. Why this rating?

23. Overall, today, how satisfied are you with your VA health care *providers* who treat your headaches?
    i. On a scale from 1-5, where 1=completely dissatisfied, 3=neither satisfied or dissatisfied, and 5=completely satisfied?
    ii. Why this rating?

Section C: Headache Condition Lifestyle Impact

Now we'd like to hear about how your headache impacts your life.

24. In what ways, would you say, your headache impacts your daily activities?

a. What do you have to change, or take into consideration, due to having headaches?
b. How is your family life impacted by your headache?
c. How are your work responsibilities impacted by your headache?
d. How are your social activities impacted by your headache?

25. How, if at all, have you experienced stigma or lack of understanding from others about your experience of headache?

    a. From whom did you experience this? (probe for during deployment and since discharge – from other military personnel at the same or higher ranks; friends; family; health care providers)

    b. How has experience of stigma related to your headache impacted your life?

Section D: SDOH issues

25. *To help us get a better understanding of any challenges related or unrelated to your headaches,* I'd like to ask about your living situation, community, and financial situation if you'd like to share. Thinking about the past 3 months, what difficulties, if any, have you experienced with paying for housing, food, transportation, medications, or other essential things in your life? Do you feel that you have supportive people to turn to?

Section 5: Recommendations/ Advice regarding Headache for Other Veterans, for providers, and for Creating Headache Centers of Excellence

26. What advice would you give to a Veteran who experiences headaches?

Section 6: Wrap- Up

27. Is there anything else you would like to share about your own experience with headache or any additional advice regarding how the VA should provide headache care?

Data Analysis Notes

Key Concepts for Analysis	
**Key Concepts**	**Interviewers' perceptions**
Any Surprises:	
Brief Summary of the Interview: 1-2 paragraphs at most.	
Important issues/points for upcoming interviews	
