# Supplementary material for: Veteran deployed burn pits exposure: headache phenotypes, trajectories, and outcomes: a mixed-methods evaluation
Source: Front Neurol. 2026 Oct 22;17:1899580. doi: 10.3389/fneur.2026.1899580 (PMC13735539; doi:10.3389/fneur.2026.1899580)
Supplement: Supplementary file 2 [file Data_Sheet_2.pdf]

21-Oct-24

Burn Pit Exposure & Headache- Veteran Survey

This study is centered at the VA National Headache Center of Excellence Research Coordination Center under the Direction of Dr. Jason Sico, National Director of Headache Center of Excellence (HCoE) Program. We aim to better understand how burn pit exposure may affect chronic health conditions. We are conducting this survey with Veterans, like yourself, to better understand your experiences with headache and other health conditions.

Completing this survey is voluntary and confidential. Results will be reported in aggregate to protect each participant's identity. Please note, you can skip questions and/or close the survey at any time. Your participation in the survey will not impact your care received at the VA. Similarly, declining to participate in the survey will not impact your VA care.

If you have any questions about this survey, please contact Dr. Nicholas Rattray Nicholas.Rattray@va.gov.

To begin, we will ask a few questions to better understand each Veteran's background. If you feel uncomfortable or unable to respond to any question in this section, you may select "prefer not to disclose" or leave a question blank.

Section 1: Sociodemographic Information

1. What term(s) best express how you describe your gender identity?
    a. Woman
    b. Man
    c. Transgender man/ female-to-male (FTM)
    d. Transgender woman/male-to-female (MTF)
    e. Non-binary
    f. Gender non-binary/gender queer/gender nonconforming
    g. Agender
    h. Bigender
    i. Other
        i. Please specify:
    j. Prefer not to disclose.
2. Do you identify as:
    a. Straight or heterosexual
    b. Lesbian, gay, or homosexual
    c. Bisexual
    d. Something else (e.g., queer, pansexual, asexual)
        i. Please specify:
    e. Don't know.
    f. Prefer not to disclose
3. What is your current relationship status?
    a. Single, never married.
    b. Married/ civil union/ domestic partnership.
    c. Living together, not married
    d. Separated
    e. Divorced
    f. Widowed
4. Please select one response to which you most closely identify for ethnicity:
    a. Hispanic or Latino
    b. Not Hispanic or Latino
    c. Unknown
    d. Prefer not to disclose.
5. Please select all responses to which you most closely identify for race:
    a. American Indian or Alaska Native
    b. Asian
    c. Black or African American
    d. Native Hawaiian or Other Pacific Islander

   e. White
   f. Unknown
   g. Prefer not to disclose.

6. How many years did you service in the military in total? Please include any time on active duty, in reserves, and/or national and coast guard within that time.

In the next section of questions, we ask about your experience during military service regarding burn pit exposure and other toxic emissions.

Section 2: Burn Pit Exposure

7. Burn pits have been a common waste disposal practice, and emissions may have health effects. To your knowledge, were you exposed to burn pits?
   a. Yes
   b. No
   c. Unsure
   d. Prefer not to disclose.
8. (If yes) Do you believe your exposure to burn pits has affected your mental or physical health?
   a. Yes, burn pit exposure affected BOTH my mental and physical health.
   b. Yes, burn pit exposure affected my mental health.
   c. Yes, burn pit exposure affected my physical health.
   d. No
   e. Unsure
   f. Prefer not to disclose.
9. Were you made aware of potential risks associated with exposure to burn pits during or after deployment?
   a. Yes
   b. No
   c. Unsure
   d. Prefer not to disclose.
10. Were you ever provided any personal protective equipment when working and/or being in proximity of burn pits?
    a. Yes
    b. No
    c. Unsure
    d. Prefer not to disclose.
11. If treatments or screening tests became available for complications arising from burn pit exposure, would you seek them out?
    a. Yes
    b. No
    c. Unsure
    d. Prefer not to disclose.
12. Did you have headache prior to being exposed to headache?
    a. Yes
    b. No
13. Following burn pit exposure, did you develop a new headache or experience exacerbation of your previous headache?

14. Are you aware of your own exposure to other potentially toxic emissions? (Other common categories include sand, dust, and particulate matter; fuel, aircraft exhaust, and other mechanical fumes; smoke from oil well fires; blast or noise.)
    a. Yes
    b. No
    c. Unsure
    d. Prefer not to disclose.
15. (If yes) Do you believe your exposure to toxic emissions has affected your mental or physical health?
    a. Yes, exposure to toxic emissions affected BOTH my mental and physical health.
    b. Yes, exposure to toxic emissions affected my mental health.
    c. Yes, exposure to toxic emissions affected my physical health.
    d. No
    e. Unsure
    f. Prefer not to disclose.
16. Were you made aware of potential risks associated with exposure to toxic emissions during or after deployment?
    a. Yes
    b. No
    c. Unsure
    d. Prefer not to disclose.
17. Were you ever provided any personal protective equipment when working and/or being in proximity of toxic emissions?
    a. Yes
    b. No
    c. Unsure
    d. Prefer not to disclose.
18. If treatments or screening tests became available for complications arising from toxic emissions, would you seek them out?
    a. Yes
    b. No
    c. Unsure
    d. Prefer not to disclose.

In the next section, we ask questions about headache symptoms and condition. Please answer as well as you can based on your health experience.

Section 3: Headache & Other Health Symptoms

19. Please specify number (#) of headache types you experience:
20. For the following questions, please consider the different types of headache you may have. Please enter the number of days in which you experience a headache in the past month, in the past 3 months, and in the past 12 months, for each type of headache listed below. If you have just one type, please enter a "0" when asked about the second type and other types of headache.

	# In Past Month	# In Past 3 Months	# In Past 12 Months
Most severe type of headache			
Second most severe type of headache			

21. About how old (in years) were you when you had your first attack of…
    a. Your most severe type of headache?
    b. Your second most severe type of headache? (if applicable)

For the following questions, consider your different types of headaches, please answer how the following statements describe your pain and other symptoms. If you have just one type of headache, answer the questions regarding the "most severe type" only.

22. Have you had at least five of these headache attacks in your lifetime?
    a. Most severe type of headache?
        i. Yes
        ii. No
    b. Second most severe type of headache?
        i. Yes
        ii. No
23. On a scale from 0-10 with 0 being no pain at all and 10 being as bad as it can be, how would you rate the pain that you have with this type of headache?
    a. Most severe type of headache?
        i. 1-10
    b. Second most severe type of headache?
        i. 1-10
24. Describe the duration of each of your headaches.
    a. Most severe type of headache?
        i. # min
        ii. # hours
        iii. # days
        iv. Headache is always there.
        v. Don't know.
    b. Second most severe type of headache?
        i. # min
        ii. # hours
        iii. # days
        iv. Headache is always there.
        v. Don't know.
25. During these headache attacks, is your skin (scalp, face, arms, etc.) sensitive to things that are not usually uncomfortable, for example, showering, shaving, combing hair, wearing glasses, or exposure to heat or cold?
    a. Most severe type of headache?
        i. Yes
        ii. No
        iii. Don't know
    b. Second most severe type of headache?
        i. Yes
        ii. No
        iii. Don't know

For the following questions, consider your different types of headaches, please answer how the following statements describe your pain and other symptoms. If you have just one type of headache, answer the questions regarding the "most severe type" only.

26. The pain is worse on just one side.
    a. Most severe type of headache?
        i. Never
        ii. Rarely
        iii. Less than half the time
        iv. Half the time or more
    b. Second most severe type of headache?
        i. Never
        ii. Rarely
        iii. Less than half the time
        iv. Half the time or more
27. The pain is pounding, pulsating, or throbbing.
    a. Most severe type of headache?
        i. Never
        ii. Rarely
        iii. Less than half the time
        iv. Half the time or more
    b. Second most severe type of headache?
        i. Never
        ii. Rarely
        iii. Less than half the time
        iv. Half the time or more
28. The pain has moderate or severe intensity.
    a. Most severe type of headache?
        i. Never
        ii. Rarely
        iii. Less than half the time
        iv. Half the time or more
    b. Second most severe type of headache?
        i. Never
        ii. Rarely
        iii. Less than half the time
        iv. Half the time or more
29. The pain is made worse by routine activities such as walking or climbing stairs.
    a. Most severe type of headache?
        i. Never
        ii. Rarely
        iii. Less than half the time

            iv. Half the time or more
    b. Second most severe type of headache?
        i. Never
        ii. Rarely
        iii. Less than half the time
        iv. Half the time or more

30. You feel nauseated or sick to your stomach
    a. Most severe type of headache?
        i. Never
        ii. Rarely
        iii. Less than half the time
        iv. Half the time or more
    b. Second most severe type of headache?
        i. Never
        ii. Rarely
        iii. Less than half the time
        iv. Half the time or more
31. Light bothers you, more than when you do not have headaches.
    a. Most severe type of headache?
        i. Never
        ii. Rarely
        iii. Less than half the time
        iv. Half the time or more
    b. Second most severe type of headache?
        i. Never
        ii. Rarely
        iii. Less than half the time
        iv. Half the time or more
32. Sound bothers you, more than when you do not have headaches.
    a. Most severe type of headache?
        i. Never
        ii. Rarely
        iii. Less than half the time
        iv. Half the time or more
    b. Second most severe type of headache?
        i. Never
        ii. Rarely
        iii. Less than half the time
        iv. Half the time or more
33. Smell bothers you, more than when you do not have headaches.
    a. Most severe type of headache?
        i. Never

   ii. Rarely
   iii. Less than half the time
   iv. Half the time or more
  b. Second most severe type of headache?
   i. Never
   ii. Rarely
   iii. Less than half the time
   iv. Half the time or more

34. You don't feel like eating.
    a. Most severe type of headache?
        i. Never
        ii. Rarely
        iii. Less than half the time
        iv. Half the time or more
    b. Second most severe type of headache?
        i. Never
        ii. Rarely
        iii. Less than half the time
        iv. Half the time or more

Visual Aura

In the next subsection, we ask questions about headache symptoms and condition related to vision. Please answer as well as you can based on your health experience.

35. Have you ever had vision changes just prior to or with any headache? Select all the apply
    a. Yes: spots, stars, lines, flashing lights, zigzag lines, or “heat waves”
    b. Yes: loss of vision or partial loss of vision
    c. No
    d. Don’t remember
36. (If yes to question above 29), how many times have you experienced these visual changes in the past 12 months?
    a. No times in the past 12 months
    b. One time
    c. Two- four times
    d. With most headaches
    e. With every headache
    f. Don’t remember
37. Have you had at least 1 headache in the last year with these visual changes?
    a. Yes
    b. No
38. How often do you have these changes in vision with your most severe type of headache?
    a. Not at all
    b. Rarely
    c. Sometimes
    d. Often
    e. All the time
39. How long do these changes in your vision last on average?
    a. Fill in number of min.
    b. N/A do not experience changes in vision
40. Have you ever had a feeling such as numbness or tingling in any part of your body or face (that was not associated with treatment) just prior to or with any headache?
    a. Yes
    b. No
    c. Don’t know
41. How many times have you experienced these sensory changes with headache in your life? Select one response
    a. No times in the past 12 months
    b. One time
    c. Two- four times
    d. With most headaches
    e. With every headache
    f. Don’t remember

Cluster Headache

Please answer the following questions about your headache experience considering ANY of your headache types.

42. Is this the worst pain you have ever experienced?
    a. Yes
    b. no
43. Imagine setting a timer. Does the headache last less than 4 hours?
    a. Yes
    b. no
44. During a headache, do one or more of these happen to you?
    a. You eye turns red on only one side
        i. Yes
        ii. no
    b. Your eye waters on only one side
        i. Yes
        ii. no
    c. Your nose runs on only one side
        i. Yes
        ii. no
    d. Your nose gets congest on only one side
        i. Yes
        ii. no

Post- Traumatic Headache

In the next section, we ask questions about if your headache symptoms and condition are related to illness or injury. Please answer as well as you can based on your health experience.

45. Did the headache start or worsen after a head injury, illness, or other event?
    a. Yes, headache started after a head injury, illness, or other event
    b. Yes, headache worsened after a head injury, illness, or other event
    c. No
46. Which do you associate with your headache?
    a. Head injury
    b. Illness
    c. Other event
        i. Please specify:
47. If yes, please check the head injury you associate with your headache?
    a. Blast injury (improvised explosive device, RPG, land mine, grenade, etc.)
    b. Blunt injury
    c. Bullet
    d. Fall
    e. Fragment
    f. Sports concussion
    g. Vehicular (any type of vehicle including airplane)
    h. Whiplash
    i. Other head injury
        i. Please specify:
48. If yes, please check the illness you associate with your headache?
    a. Dental problems
    b. Infection
    c. Heart or vascular disorders
    d. Lack of oxygen
    e. Mental health problems
    f. Pregnancy-related illness
    g. Substance use or withdrawal
    h. Thyroid disease
    i. Other illness
        i. Please specify:
49. How long ago did the head injury, illness, or event occur?
    a. Within the past month
    b. 1-3 months
    c. 3-6 months
    d. 6-12 months
    e. >1 year
50. How long after the head injury, illness, or event did the headache start?

a. Immediately
b. Within 7 days
c. 7-30 days
d. 1-4 months
e. 3-6 months
f. 6-12 months
g. >1 year

Section 4: Headache Outcome

In the next section, we ask questions about how your headache symptoms and condition impact daily life. Please answer as well as you can based on your health experience.

51. When you have headaches, how often is the pain severe?
    a. Never
    b. Rarely
    c. Sometimes
    d. Very Often
    e. Always
52. How often do headaches limit your ability to do usual daily activities including household work, work, school, or social activities?
    a. Never
    b. Rarely
    c. Sometimes
    d. Very Often
    e. Always
53. When you have a headache, how often do you wish you could lie down?
    a. Never
    b. Rarely
    c. Sometimes
    d. Very Often
    e. Always
54. In the past 4 weeks, how often have you felt too tired to do work or daily activities because of your headaches?
    a. Never
    b. Rarely
    c. Sometimes
    d. Very Often
    e. Always
55. In the past 4 weeks, how often have you felt fed up or irritated because of your headaches?
    a. Never
    b. Rarely
    c. Sometimes
    d. Very Often
    e. Always
56. In the past 4 weeks, how often did headaches limit your ability to concentrate on work or daily activities?
    a. Never
    b. Rarely
    c. Sometimes
    d. Very Often

e. Always

Section 5: Functional Status/Quality of Life

The following questions will ask about current quality of life and health impact. Please answer as well as you can based on your current experience.

Sleep Quality Scale

57. During the **past week**, how would you rate your sleep quality overall?
    a. 0 = terrible
    b. 1-3 = poor
    c. 4-6 = fair
    d. 4-9 = good
    e. 10 = excellent

Pain, Enjoyment, General activities (PEG)

58. What number best describes your pain on average in the past week?
    - 0- no pain
    - 1
    - 2
    - 3
    - 4
    - 5
    - 6
    - 7
    - 8
    - 9
    - 10- pain as bad as you can imagine
59. What number best describes how, during the past week, pain has interfered with your enjoyment of life?
    - 0- no pain
    - 1
    - 2
    - 3
    - 4
    - 5
    - 6
    - 7
    - 8
    - 9
    - 10- pain as bad as you can imagine
60. What number best describes how, during the past week, pain has interfered with your general activity?

- <u>0- no pain</u>
- <u>1</u>
- <u>2</u>
- <u>3</u>
- <u>4</u>
- <u>5</u>
- <u>6</u>
- <u>7</u>
- <u>8</u>
- <u>9</u>
- <u>10- pain as bad as you can imagine</u>

(Pain self-efficacy: PSEQ-2)
Please rate how confident you are that you can do the following things, despite the pain. Answer choices: 0 = not at all confident, 6 = completely confident

61. I can do some form of work, despite the pain (work includes housework and paid and unpaid work)
    a. 0=not at all confident
    b. 1
    c. 2
    d. 3
    e. 4
    f. 5
    g. 6= completely confident
62. I can live a normal lifestyle, despite the pain
    a. 0=not at all confident
    b. 1
    c. 2
    d. 3
    e. 4
    f. 5
    g. 6= completely confident

<u>PHQ2 & GAD2</u>

(PHQ2 and GAD2): Over the **<u>past two weeks,</u>** how often have you been bothered by any of the following problems? Answer choices: 0 = Not at all, 1 = Several days, 2 = more than half the days, 3 = nearly every day

63. Feeling down, depressed, or hopeless
    a. 0= not at all
    b. 1= several days
    c. 2= more than half the days

   d. 3= nearly everyday

64. Little interest or pleasure in doing things
    a. 0= not at all
    b. 1= several days
    c. 2= more than half the days
    d. 3= nearly everyday
65. Feeling nervous, anxious, or on edge
    a. 0= not at all
    b. 1= several days
    c. 2= more than half the days
    d. 3= nearly everyday
66. Not being able to stop or control worry
    a. 0= not at all
    b. 1= several days
    c. 2= more than half the days
    d. 3= nearly everyday

VR-12

The following questions ask for your views about your health- how you feel and how well you are able to do your usual activities. If you are unsure how to answer a question, please choose the answer that best fits your life right now.

67. In general, would you say your health is
    a. Excellent
    b. Very good
    c. Good
    d. Fair
    e. Poor

Now, we'd like to ask you some questions about how your health may have changed.

68. Compared to one year ago, how would you rate your physical health in general now?
    a. Much better
    b. Slightly better
    c. About the same
    d. Slightly worse
    e. Much worse
69. Compared to one year ago, how would you rate your emotional problems (such as feeling anxious, depressed, or irritable) now?
    a. Much better
    b. Slightly better
    c. About the same
    d. Slightly worse
    e. Much worse

Section 6: Social Determinants of Health

The following questions will ask about any unmet needs you might have. Please answer as well as you can based on your current experience.

70. The past three months, did you ever run out of food and you were not able to access more food or have the money to buy more food?
    a. Yes
    b. No
71. Do you currently have any concerns about having enough food?
    a. Yes
    b. No
72. In the past two months, have you been living in stable housing that you own, rent, or stay in as part of a household?
    a. Yes
    b. No
73. Are you worried or concerned that in the next two months that you may NOT have stable housing that you own, rent, or stay in as part of a household?
    a. Yes
    b. No
74. Are you currently without a place to stay?
    a. Yes
    b. No
75. Do you have trouble paying for your utilities (i.e., gas, electricity, phone, and water)?
    a. Yes
    b. No
76. Has the electric, gas, oil, or water company threatened to shut off services in your home?
    a. Yes
    b. No
77. Has lack of transportation kept you from medical appointments, meetings, work, or from getting things needed for daily living?
    a. Yes
    b. No
78. Please select the answer that most represents your current experience. How often do you feel lonely or isolated from those around?
    a. Never
    b. Rarely
    c. Sometimes
    d. Often
    e. Always

79. When you consider your household income from all sources today (including disability or social security), which statement best describes how you feel about your financial situation?

    a. Comfortable
    b. Just enough to make ends meet
    c. Not enough to make ends meet
    d. Prefer not to say

Section 7: Satisfaction, Care Seeking, & Patient Acceptability

In the next section, we ask questions about your healthcare experience and satisfaction. Please answer as well as you can based on your health experience.

80. Overall, how satisfied are you with your headache care at the VA on a scale of 1 to 5 (where 1 is not at all satisfied and 5 is very satisfied?
    a. 1
    b. 2
    c. 3
    d. 4
    e. 5
81. In the past 12 months, have you received headache care outside the VA?
    a. Yes
    b. No
82. Overall, how satisfied are you with your VA care on a scale of 1 to 5 (where 1 is not at all satisfied and 5 is very satisfied?
    a. 1
    b. 2
    c. 3
    d. 4
    e. 5

Section 8: Optional Follow- Up Interview

We would like to learn more from you about your experience with headache and care.

Would you be interested in completing a follow-up, virtual, qualitative interview with our team to help us better understand the impact of burn pit exposure and headache condition on Veterans such as yourself?

> Please note that even after providing your name and contact information below, your survey responses will remain anonymous. Also, if you change your mind about participating in an interview, when you are contacted by our team, you can decline to schedule an interview.
>
> Whether you participate in an interview or not will not in any way affect the care you receive from the VA.

83. Please provide your name:
84. Please provide your phone number:
85. Please provide your preferred email:
86. Do you prefer text, call, or email?

Thank you for your service to our country and for your time today. If you have any questions about this survey, please contact Dr. Nicholas Rattray (Nicholas.Rattray@va.gov).
