# Supplementary material for: Veteran deployed burn pits exposure: headache phenotypes, trajectories, and outcomes: a mixed-methods evaluation
Source: Front Neurol. 2026 Oct 22;17:1899580. doi: 10.3389/fneur.2026.1899580 (PMC13735539; doi:10.3389/fneur.2026.1899580)
Supplement: Supplementary file 3 [file Data_Sheet_3.pdf]

Supplementary Materials S1. Headache Outcomes by Reported Open Burn Pit Exposure

Panel A. Statistical Comparisons						
Outcome	Primary analysis, p	Unknown classified as exposed, p	Unknown classified as not exposed, p	Primary MD (95% CI)	Unknown classified as exposed MD (95% CI)	Unknown classified as not exposed MD (95% CI)
HIT-6	0.047	0.077	0.032	3.26 [0.04, 6.48]	2.99 [-0.32,6.30]	2.82 [0.25, 5.39]
ISI	0.004	0.009	<.001	3.60 [1.15, 6.04]	3.33 [0.86, 5.81]	2.91 [1.28, 4.54]
PEG	0.051	0.068	0.027	0.97 [-0.01, 1.94]	0.91 [-0.07, 1.88]	0.73 [0.08, 1.37]
PHQ	0.246	0.309	0.053	0.45 [-0.31, 1.22]	0.39 [-0.37, 1.15]	0.50 [0.01,1.00]
GAD	0.080	0.108	0.017	0.72 [-0.09, 1.54]	0.66 [-0.15, 1.46]	0.65 [0.12, 1.18]
PSEQ	0.589	0.645	0.337	-0.34 [-1.60, 0.91]	-0.29 [-1.55, 0.96]	-0.41 [-1.25, 0.43]
Frequency	0.252	0.315	0.046	16.82 [-12.40, 46.04]	14.31 [-14.16, 42.78]	21.15 [0.42,41.88]

Panel B. Descriptive Statistics				
Outcome	Primary analysis with exposure, mean (SD)	Primary analysis without exposure, mean (SD)	Unknown classified as exposed, mean (SD)	Unknown classified as not exposed, mean (SD)

HIT-6	61.47 (7.61)	58.21 (8.93)	61.20 (7.90)	58.65 (9.41)
ISI	15.47 (5.90)	11.88 (5.28)	15.21 (6.00)	12.56 (6.00)
PEG	5.08 (2.31)	4.11 (2.66)	5.02 (2.32)	4.35 (2.50)
PHQ	2.28 (1.82)	1.83 (1.93)	2.23 (1.82)	1.79 (1.82)
GAD	2.60 (1.96)	1.88 (1.78)	2.53 (2.33)	1.95 (1.79)
PSEQ	7.99 (2.96)	8.33 (3.33)	8.04 (2.99)	8.40 (3.22)
Frequency	47.84 (133.30)	31.02 (55.10)	45.33 (127.2)	26.69 (49.24)
