# Supplementary material for: Veteran deployed burn pits exposure: headache phenotypes, trajectories, and outcomes: a mixed-methods evaluation
Source: Front Neurol. 2026 Oct 22;17:1899580. doi: 10.3389/fneur.2026.1899580 (PMC13735539; doi:10.3389/fneur.2026.1899580)
Supplement: Supplementary file 4 [file Data_Sheet_4.pdf]

Supplementary Materials S3. Effects of headache phenotype on health outcomes: A series of four-way analysis of variance (ANOVA) tests and Post Hoc Comparisons

Outcome	Overall p	Pairwise Comparison	Mean Difference	95% CI	Adjusted p	Post Hoc Test
Headache Impact Test	<.001	Migraine vs ‘Other Headache’	6.15	[3.4,8.9]	<.001	Tukey-Kramer
		Migraine vs PTH	-1.39	[1.1, -3.9]	.467	Tukey-Kramer
		Migraine vs Cluster Headache	0.35	[-5.7,6.4]	.999	Tukey-Kramer
		PTH vs ‘Other Headache’	7.54	[4.8,10.3]	<.001	Tukey-Kramer
		PTH vs Cluster Headache	1.75	[-4.3,7.7]	.876	Tukey-Kramer
		Cluster Headache vs ‘Other Headache’	5.80	[-0.3,11.9]	.072	Tukey-Kramer
Pain, Enjoyment of life, and General activity	.013	Migraine vs ‘Other Headache’	0.65	[-0.2,1.5]	.230	Tukey-Kramer
		Migraine vs PTH	-0.47	[-1.3,0.3]	.425	Tukey-Kramer
		Migraine vs Cluster Headache	-0.13	[-2.0,1.7]	1.00	Tukey-Kramer
		PTH vs ‘Other Headache’	1.12	[0.3,2.0]	.006	Tukey-Kramer
		PTH vs Cluster Headache	0.34	[-1.5,2.2]	.966	Tukey-Kramer
		Cluster Headache vs ‘Other Headache’	0.78	[-1.1,2.7]	.709	Tukey-Kramer
Pain Self-Efficacy Questionnaire	.028	Migraine vs ‘Other Headache’	-	-	.015	DSCF
		Migraine vs PTH	-	-	.969	DSCF
		Migraine vs Cluster Headache	-	-	1.00	DSCF

		PTH vs 'Other Headache'	-	-	.108	DSCF
		PTH vs Cluster Headache	-	-	1.00	DSCF
		Cluster Headache vs 'Other Headache'	-	-	.538	DSCF
Insomnia Severity Index	<.001	Migraine vs 'Other Headache'	2.93	[0.7,5.1]	.004	Tukey-Kramer
		Migraine vs PTH	-0.64	[-2.6,1.4]	.841	Tukey-Kramer
		Migraine vs Cluster Headache	0.35	[-4.4,5.1]	.998	Tukey-Kramer
		PTH vs 'Other Headache'	3.57	[1.4,5.8]	<.001	Tukey-Kramer
		PTH vs Cluster Headache	0.99	[-3.7,5.7]	.948	Tukey-Kramer
		Cluster Headache vs 'Other Headache'	2.58	[-2.2,7.4]	.505	Tukey-Kramer
Patient Health Questionnaire	.005	Migraine vs 'Other Headache'	-	-	.140	DSCF
		Migraine vs PTH	-	-	.384	DSCF
		Migraine vs Cluster Headache	-	-	.998	DSCF
		PTH vs 'Other Headache'	-	-	.002	DSCF
		PTH vs Cluster Headache	-	-	.842	DSCF
		Cluster Headache vs 'Other Headache'	-	-	.882	DSCF
Generalized Anxiety Disorder	.008	Migraine vs 'Other Headache'	-	-	.014	DSCF
		Migraine vs PTH	-	-	1.00	DSCF

		Migraine vs Cluster Headache	-	-	.922	DSCF
		PTH vs ‘Other Headache’	-	-	.009	DSCF
		PTH vs Cluster Headache	-	-	.918	DSCF
		Cluster Headache vs ‘Other Headache’	-	-	.876	DSCF
Frequency	<.001	Migraine vs ‘Other Headache’	-	-	<.001	DSCF
		Migraine vs PTH	-	-	.999	DSCF
		Migraine vs Cluster Headache	-	-	.948	DSCF
		PTH vs ‘Other Headache’	-	-	<.001	DSCF
		PTH vs Cluster Headache	-	-	.948	DSCF
		Cluster Headache vs ‘Other Headache’	-	-	.004	DSCF
